# The optimization of poly(vinyl)-alcohol-alginate beads with a slow-release compound for the aerobic cometabolism of chlorinated aliphatic hydrocarbons†

**DOI:** 10.1039/d3su00409k

**Published:** 2024-03-14

**Authors:** Conor G. Harris, Hannah K. Gedde, Audrey A. Davis, Lewis Semprini, Willie E. Rochefort, Kaitlin C. Fogg

**Affiliations:** a School of Chemical, Biological, and Environmental Engineering, Oregon State University Corvallis OR 97331 USA +541-737-1777 kaitlin.fogg@oregonstate.edu

## Abstract

Chlorinated aliphatic hydrocarbons (CAHs), such as *cis*-1,2-dichloroethylene (*c*DCE), are prevalent in groundwater at many locations throughout the United States. When immobilized in hydrogel beads with slow-release compounds, the bacteria strain *Rhodococcus rhodochrous* ATCC 21198 can be used for the *in situ* bioremediation of *c*DCE. These hydrogel beads must exhibit high mechanical strength and resist degradation to extend the lifetime of slow-release compounds and bioremediation. We engineered poly(vinyl)-alcohol – alginate (PVA-AG) beads to immobilize ATCC 21198 with the slow-release compound, tetrabutoxysilane (TBOS) that produces 1-butanol as a growth substrate, for high mechanical strength. We optimized three inputs (concentration of PVA, concentration of AG, and the crosslinking time) on two responses (compressive modulus and rate of oxygen utilization) for batch incubation experiments between 1 and 30 days using a design of experiments approach. The predictive models generated from design of experiments were then tested by measuring the compressive strength, oxygen utilization, and abiotic rates of hydrolysis for a predicted optimal bead formulation. The result of this study generated a hydrogel bead with immobilized *R. rhodochrous* ATCC 21198 and TBOS that exhibited a high compressive modulus on day 1 and day 30, which was accurately predicted by models. These hydrogel beads exhibited low metabolic activity based on oxygen rates on day 1 and day 30 but were not accurately predicted by the models. In addition, the ratio between oxygen utilization and abiotic rates of hydrolysis were observed to be roughly half of what was expected stoichiometrically. Lastly, we demonstrated the capability to use these beads as a bioremediation technology for *c*DCE as we found that, for all bead formulations, *c*DCE was significantly reduced after 30 days. Altogether, this work demonstrates the capability to capture and enhance the material properties of the complex hydrogel beads with predictive models yet signals the need for more robust methods to understand the metabolic activity that occurs in the hydrogel beads.

## Abstract 2

Poly(vinyl)-alcohol – alginate beads with immobilized cells and a slow-release compound were optimized for long-term bioremediation of chlorinated solvents.

## Graphical abstract

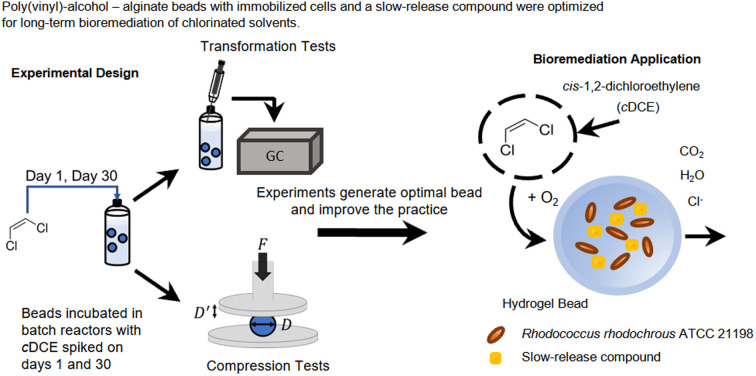

**Sustainability spotlight**

Chlorinated volatile organic compounds (VOCs) are one of the most commonly detected pollutants in public supply wells and pose a threat to human and environmental health. Chlorinated VOCs have diffused into saturated low permeability zones within aquifers that require long-term passive techniques. Our work explores the long-term passive and economical remediation technique of *in situ* aerobic cometabolic bioremediation with immobilized bacteria and slow-release compounds in hydrogel beads. This work specifically evaluates and optimizes the material properties of the hydrogel bead based on the mechanical strength of the bead and the oxygen utilization of the microbe to extend the time for bioremediation. Our work aligns with the UN sustainable development goals: Clean Water and Sanitation^6^ and Industry, Innovation, and Infrastructure.^9^

## 1. Introduction

A subset of chlorinated aliphatic hydrocarbons (CAHs) persist as contaminants in groundwater aquifer systems.^1–3^ Actually, these chlorinated hydrocarbon compounds are some of the most common groundwater contaminants globally.^4^ One of the most abundant CAHs found in groundwater is *cis*-1,2-dichloroethylene (*c*DCE).^5^ Currently, the EPA regulates *c*DCE at a maximum contaminant level goal and maximum contaminant level (MCL) of 0.07 mg L^−1^ due to potential health effects such as liver and kidney problems, drowsiness and nausea, and cardiovascular problems.^6^ The release of *c*DCE into the environment has propagated from the use of chloroethylenes as solvents and synthetic feedstocks.^7^ Specifically, groundwater contamination by *c*DCE occurs due to the reductive dechlorination of more highly chlorinated compounds, such as tetrachloroethylene and trichloroethylene.^6^ When aquifers lack *Dehalococcoides* or *Dehalogenimonas* microbes that can reduce dichloroethylenes (DCEs) to ethene, DCEs accumulate in the groundwater, known as a DCE stall.^8–10^ Instead, the process of aerobic cometabolism generates metabolites of epoxides and alcohols that can be degraded into carbon dioxide and can degrade contaminants to concentrations below the thresholds needed to support direct metabolism.^8^ This study focused on the bacterium *Rhodococcus rhodochrous* ATCC 21198 (ATCC 21198) that has been previously validated to aerobically cometabolize a wide range of CAHs and 1,4-dioxane, which was used as a stabilizer in CAHs and is typically found as a co-contaminant where CAHs are measured.^11,12^ Therefore, it is advantageous to treat such zones *in situ* with the use of aerobic cometabolic bioremediation.^13^

Currently, the most common implementation of bioremediation for CAHs is biostimulation, where the induction of an enzyme occurs through the continuous sparging of a gaseous substrate.^14^ Either native microbes capable of aerobic cometabolism exist in aquifer systems or aquifers receive injections of non-native microbes, known as bioaugmentation. While sparging can stimulate or enhance aerobic biodegradation by microorganisms, the volatilization of contaminates can occur and present hazardous atmospheres.^15^ In contrast, permeable reactive bio-barriers are *in situ* permeable treatment zones that contain materials to enhance or stimulate microorganisms to transform contaminants.^16^ These permeable reactive bio-barriers have the potential to reduce costs and eliminate the need for continuous injections of substrates.^17^

We and others have successfully developed passive treatment systems that can be used in permeable reactive bio-barriers to transform CAHs.^18,19^ Specifically, we developed a passive cometabolic system consisting of gellan gum hydrogel beads with immobilized ATCC 21198 and a slow-release compound, tetrabutoxysilane (TBOS), that hydrolyzes gradually over time to generate 1-butanol, a carbon source used to stimulate ATCC 21198.^20^ We demonstrated the use of the gellan gum immobilization system in column studies, with results of more than 99% removal of *c*DCE and with 1-butanol concentrations below detection at a hydraulic residence time of one day.^12^ While this system was successful in demonstrating the capability to encapsulate both ATCC 21198 and a slow-release compound, the gellan gum hydrogel beads exhibited poor mechanical strength and degraded rapidly, greatly reducing the permeability of the column packing. This presented a challenge, as the hydrogel provides protection to the immobilized bacteria against the harsh soil macroenvironment, helping to extend the metabolic activity of cells.^21^ Consequently, hydrogels must resist compression from the weight of the packed column and remain intact such that the packed column maintains a high permeability to allow contaminants to flow through and into beads. Therefore, the hydrogel beads designed for permeable bio-barriers must be engineered with high mechanical strength.

Poly(vinyl)-alcohol (PVA) is a synthetic, yet biocompatible polymer that is resistant to degradation and has high mechanical strength.^22–24^ Alginate (AG) is a natural polysaccharide produced from brown algae that is highly biocompatible and is frequently used in biomedical applications.^25^ Many crosslinking techniques exist for both PVA and AG, however with the long-term goal of mass production of these hydrogel beads, this study focuses on chemical crosslinking where the polymer linkage occurs through the introduction of free ions to polymer solutions. Boric acid forms bonds with the oxygen groups occurring on the diol linkages in PVA, whereas divalent cations, such as calcium chloride, bind AG between its polymeric α-l-guluronate units. Hydrogels formed from both PVA and AG create semi-interpenetrating polymer networks, where the polymer chains interlace between each other but do not bond.^22,24^ Together, PVA-AG hydrogels offer wide applicability and possess key characteristics that enable the entrapment of whole cells.^26–29^

In this study, we optimized PVA-AG hydrogel beads to immobilize ATCC 21198 and TBOS with high compressive moduli. The optimization objectives were to simultaneously maximize the mechanical strength of the hydrogel bead and minimize the rate of substrate utilization based on oxygen utilization over the course of 30 days. This was attempted using Design of Experiments (DOE), a powerful statistical optimization technique that provided the experimental design to empirically model and optimize the immobilization method while reducing the number of experiments required compared to a traditional scientific method approach. Using DOE, we generated predictive models of the output variables measured as a function of the input variables. The predictive models were then tested by measuring the compressive strength, oxygen utilization, and abiotic rates of hydrolysis for a predicted optimal bead formulation.

## 2. Materials and methods

### 2.1. Cell culture

Dr Michael Hyman (NC State) provided us with ATCC 21198. Additionally, the American Type Culture Collection (ATCC) (Manassas, Virginia, United States) maintains a commercial stock of ATCC 21198. Details on growth of ATCC 21198 in batch reactors for immobilization in PVA-AG hydrogel beads are provided in the ESI (Section ESI1–3†).

### 2.2. Immobilizing ATCC 21198 and TBOS with PVA – AG beads

**Fig. 1:**
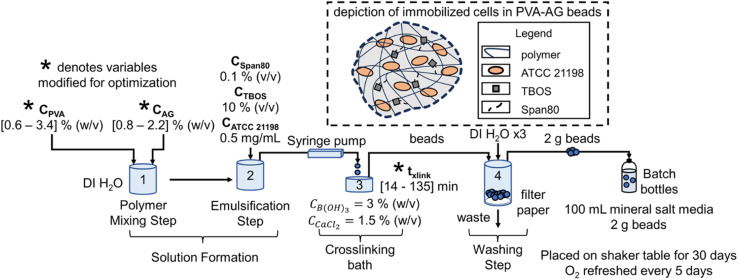
Immobilization method to bead performance batch testing process flow diagram, including a depiction of the immobilized cells in PVA-AG beads. Here *C*_PVA_ is the concentration of PVA, *C*_AG_ is the concentration of alginate, DI H_2_O is deionized water, *C*_Span80_ is the concentration of Span80, *C*_TBOS_ is the concentration of TBOS, *C*_ATCC 21198_ is the concentration of ATCC 21198, *C*_B(OH)_3__ is the concentration of boric acid in the crosslinking bath, *C*_CaCl_2__ is the concentration of calcium chloride in the crosslinking bath, and *t*_*x*link_ is the crosslinking time.

ATCC 21198 was immobilized with a slow-release compound in PVA-AG hydrogel beads using an entrapment method, where cells are trapped in the pores of crosslinked polymers (Fig. 1).^30,31^ 99+% hydrolyzed PVA (molecular weight distribution: 85–124 kDa) (Sigma-Aldrich St. Louis, Missouri, United States) solutions were prepared by dissolving the appropriate amount of PVA in autoclaved water at 80 °C. Sodium alginate (Cape Crystal Brands, Summit, New Jersey, United States) solutions were prepared by dissolving the appropriate amount of sodium alginate in autoclaved water at room temperature. Using a 4-Bladed, IKA 0741300 Propeller Stirrer (Cole-Parmer, Vernon Hills, Illinois, United States) attached to an IKA RW 20 Digital Dual-Range mixer (Cole-Parmer), the polymer solutions, Span80 (Ohio Valley Specialty Company, Marietta, Ohio, United States), tetrabutoxysilane (TBOS) (90% purity) (United Chemical Technologies, Levittown, Pennsylvania, United States), and suspended ATCC 21198 (in its late exponential growth state) were mixed until the solution was emulsified and homogeneous. Note, for optimal beads, we used higher purity TBOS (98% purity) (VWR International, Radnor, Pennsylvania, United States). The final suspension of cells contained [0.6–3.4] % (w/v) PVA, [0.8–2.2] % (w/v) AG, 0.1% (v/v) Span80, 10% (v/v) TBOS, and 0.5 mg mL^−1^ of ATCC 21198. The concentrations of PVA and AG were selected based on their capability to crosslink and the total viscosity of the solution. Below 0.6 and 0.8% (w/v) of PVA and AG, respectively, gels would not form; yet at concentrations higher than 3.4 and 2.2% (w/v) of PVA and AG, respectively, the mixed polymer solution would be too difficult to extrude. 10% (v/v) was used for TBOS as this was determined by preliminary studies as a high concentration that could be successfully emulsified into all pre-gel solutions. Span80 is a nonionic surfactant used to emulsify TBOS into the pre-gel solution with polymers and cells. The amount of cell inoculum was added to beads with the intention to grow cells in the hydrogel beads during a 30 day incubation period.

Beads were generated using a IPS-14S syringe pump (Inovenso Technology Inc, Cambridge, Massachusetts, United States) to extrude the polymer solution dropwise through an 16-gauge metal blunt point needle (Hamilton Company, Reno, Nevada, United States) into a crosslinking bath comprised of approximately 100 mL of 3% (w/v) boric acid (Honeywell International Inc., Charlotte, North Carolina, United States) and 1.5% (w/v) calcium chloride (Merck KGaA, Darmstadt, Germany) in a 150–250 mL Pyrex beaker with a magnetic stir rod placed on a magnetic stir plate. To reduce the degree of elongation in beads, we adjusted the solution flow rate between 10 and 20 mL h^−1^. To crosslink the beads, they remained in the crosslinking bath between 14 and 135 min, timed from when the last bead dropped into solution. After crosslinking, the crosslinking solution with beads was poured into a Coors Buchner funnel laid with qualitative Grade 1 filter paper (Whatman, Maidstone, United Kingdom). They were then washed with deionized water up to 3 times under vacuum at approximately 15 inHg of vacuum using a vacuum filtering side-arm flask attached to a lab vacuum spigot using vacuum tubing.

### 2.3. Incubation of immobilized ATCC 21198 in 150 mL batch reactors

Batch reactors were used to evaluate both the performance of the cometabolic transformation by immobilized ATCC 21198 with TBOS and the durability of the hydrogel beads at days 1 and 30. The two time points, days 1 and 30, were used to evaluate the performance of beads to select a bead formula for column studies that can last up to two years. Batch reactors consisted of 125 mL Wheaton serum bottles (DWK Life Sciences Wheaton, Stoke-on-Trent, United Kingdom) with nominal volumes of 155 mL sealed with screw on caps fitted with gray butyl rubber septa as were previously described in Rasmussen *et al.*^20^ Batch reactors contained 100 mL of phosphate-buffered 1X MSM with an addition of immobilized cells to achieve an initial cell concentration of 1 mg cells per L which was 2 g of beads per batch bottle. The headspace of the batch reactor (55 mL) contained air as the source of oxygen. Batch reactors were mixed constantly on a New Brunswick Scientific G10 Gyratory shaker table (Eppendorf, Hamburg, Germany) at 100 RPM at 20 °C.

### 2.4. Cometabolic and metabolic analytical methods

The performance of cometabolism by immobilized strain ATCC 21198 was evaluated by using *c*DCE as a surrogate for cometabolism based on the ability of ATCC 21198 to cometabolize *c*DCE, as well as 1,1,1-TCA and 1,4-D that was observed in past studies.^20^ This is due to the fast rates of *c*DCE transformation compared to other CAHs observed in prior cometabolic transformation experiments when using ATCC 21198.^12,20^ The reactors used for metabolic and cometabolic transformation experiments received additions of environmentally relevant aqueous concentrations (250 ppb) of *c*DCE (Tokyo Chemical Industry, Tokyo, Japan) at days 1 and 30 after beads were added to batch reactors. Oxygen served as the electron accepter for the metabolic utilization of 1-butanol product from TBOS and the cometabolic transformation. Batch reactors received oxygen every 5 days by opening the reactors in a sterile biosafety cabinet to replenish oxygen used in metabolism through the exchange of air to the batch reactor headspace. *c*DCE was not present when batch reactors were opened.

We measured volatile compounds in batch reactors as was previously described by Rasmussen *et al.*^20^ Samples of headspace were taken from batch reactors using a 100 μL Hamilton Gastight Syringe and were injected into either a 5890 or 6890 Series HP Gas Chromatograph (GC). 5890 Series HP GCs was equipped with a thermal conductivity detector to measure O_2_, whereas the 6890 Series HP GC was equipped an electron capture detector to measure *c*DCE. See gas chromatography details in the ESI (Section ESI4†) The total mass (*m*_T_) in the batch reactors was calculated as follows:1
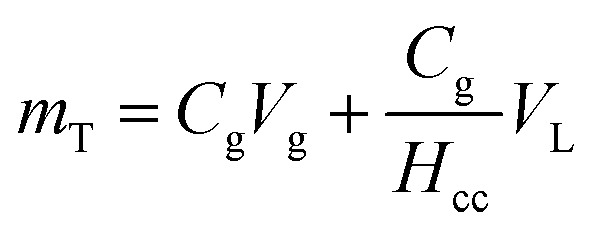
where *C*_g_ is the concentration of compound in the headspace, *V*_g_ is the volume of headspace in the batch reactor, *V*_L_ is the volume of liquid in the batch reactor, and *H*_cc_ is the Henry's Law Constant.

*H*_CC_ values, taken as the dimensionless ratio between gas- and aqueous-phase concentration were 31.5 and 0.16 for O_2_ and *c*DCE, respectively at 20 °C.^32,33^*m*_T_ values were normalized with negative control batch reactors to remove variability in instrument measurements. Normalization was applied by dividing the negative control total mass *m*_T−_ by the initial mass at time, *t* = 1 [d], *m*_T−,0_, such that 
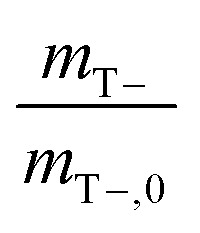
, and multiplying the inverse of this value, 
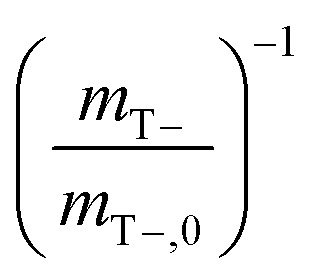
, to active batch reactor *m*_T_ values. The values of 
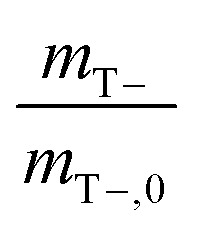
 ranged from 0.63 to 1.2, and 0.78 to 1.1 for O_2_ and *c*DCE, respectively. Negative control batch reactors consisted of batch reactors with 3 mL of 2% (w/v) sodium azide injected into medium to inhibit microbial activities.

Zero-order rate laws applied to the total mass measured over time, *t*, were used to obtain the rates for *c*DCE (*k*_*c*DCE_) and oxygen (*k*_O_2__). Sample data is provided in the ESI (Section ESI5: Fig. S1A and B†). Further, rates were normalized by the weight of the beads added to batch reactors to account for the cells that can grow within the beads, such that:2
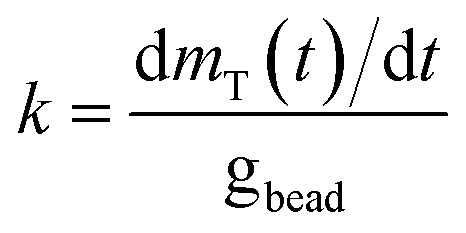
where d*m*_T_(*t*)/d*t* is the change of mass over time, and g_bead_ is the weight of the beads added to batch reactors.

A transformation yield, *T*_Y_, was calculated for days 1 and 30 as ratio between the amount of *c*DCE transformed and the amount of O_2_ consumed to provide how efficient the cells immobilized in different hydrogel formulations were with respect to the primary substrate, 1-butanol, such that:3
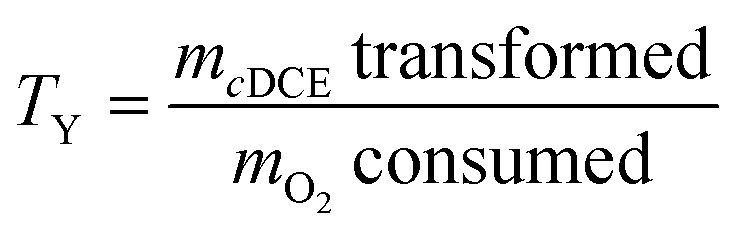
where *m*_*c*DCE_ is the mass of *c*DCE transformed and *m*_O_2__ is the mass of O_2_ consumed at 1 or 30 days.

Paired *t*-tests were used to evaluate the significance between rates and the transformation yields at days 1 and 30.

### 2.5. Unconfined compression tests for individual beads

The durability of individual beads in batch bottles between 1 and 30 days was evaluated using unconfined compression tests. For these batch reactors, we decided against injecting *c*DCE to remove potential hazards with handling *c*DCE. Unconfined compression tests on an ARG2 rheometer (TA Instruments, New Castle, Delaware, United States) were used to obtain compressive modulus data for individual beads. Individual beads were centered on the bottom Peltier plate and the top 25 mm diameter plate was lowered until contact was achieved with the bead such that the normal force read 0.01 N. The gap at initial contact was set as the undeformed diameter, *D*, of each bead. The compression of all beads proceeded at a constant strain rate (*ε* = 0.005 s^−1^) while collecting the resultant normal force data and the axial displacement data. Termination of the procedure occurred once the gap fell below 50 μm to obtain the entire stress–strain curve. For all measurements, beads were irreversibly deformed during the experiment (Section ESI6: Fig. S2†).

The compressive modulus refers to the stiffness of a material determined from the slope in the linear region of a stress–strain curve obtained from compression tests. We used the stress–strain relationship derived by Hertz for the compressive modulus for spherical particles: as a function of the force *F* and the deformation Δ*D*:^34^4
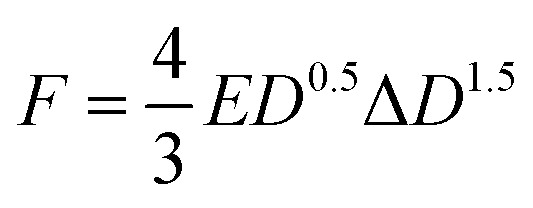
where *F* is the force applied on the sample, *E* is the compressive modulus, *D* is the initial undeformed diameter, and Δ*D* is defined as follows:

5 Δ*D* = *D* − *D*′

where *D* is the initial undeformed diameter and *D*′ is the deformed diameter due to *F*.

Typical stress–strain data for hydrogel beads is included in the ESI (Section ESI7: Fig. S3†). The elastic loss (Δ*E*) refers to the percent loss of the compressive moduli between day 1 and day 30 used to quantify the degradability of hydrogel beads:6
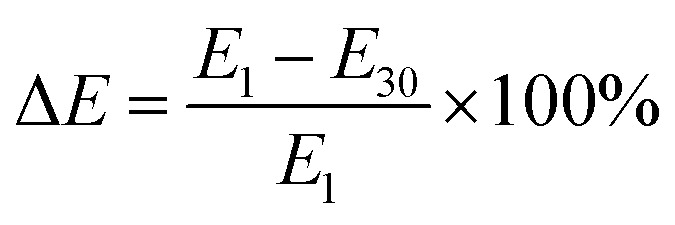
where *E*_1_ is the compressive moduli on day 1 and *E*_30_ is the compressive moduli on day 30. Elastic loss translates to a degree of degradation of hydrogels as a decrease in the compressive moduli between day 1 and day 30 would result from the dissociation of ionically crosslinked polymer chains.

Paired *t*-tests were used to evaluate the significance between rates at day 1 and day 30.

### 2.6. Performance evaluation for the abiotic hydrolysis of TBOS

The rates of hydrolysis of TBOS were determined using methods described in Rasmussen *et al.*^20^ 3 mL of 2% (w/v) sodium azide (Spectrum Chemical, Gardena, California, United States) was added into batch reactors to poison cells and to stop the utilization of the hydrolysis product, 1-butanol. The complete hydrolysis of TBOS is as follows as we previously described in Vancheeswaran *et al.*:^35^

7 Si(OC_4_H_7_)_4_ + 4H_2_O → 4C_4_H_8_ + Si(OH)_4_

### 2.7. Abiotic hydrolysis analytical methods

The release of 1-butanol (1-BuOH) was used to determine the rate of hydrolysis from TBOS. Samples were prepared for injection using a Tekmar Lumin heated purge and trap concentrator with an Aqua Tek 70 Liquid Autosampler (Teledyne Tekmar, Mason, Ohio, United States). Samples were injected into a HP 6890 Series Gas Chromatograph with a 5973 Agilent Mass Selective Detector (GC-MS). The GC-MS was fitted with a Restek Rtx-VMS capillary column (Restek, Bellefonte, Pennsylvania, United States) to separate compounds. The GC-MS received injections of diluted aqueous samples from batch bottles while in the single-ion monitoring mode for *m*/*z* of 56 to measure 1-BuOH in the samples.

### 2.8. Central composite orthogonal design

A Design of Experiments (DOE) statistical method was used to design experiments due to its prevalence of use in biomaterials research and due to the successful optimization of other immobilization methods using DOE.^28,36,37^ The application of a central composite orthogonal design (CCO) established the experimental matrix for this study. Details on the DOE method are provided in the ESI (Section ESI8†). The concentration of PVA *C*_PVA_ [% (w/v)], the concentration of alginate *C*_AG_ [% (w/v)], and cross-linking time *t*_*x*link_ [min] were set as the three independent variables. The variables *C*_PVA_, *C*_AG_, and *t*_*x*link_ are coded by the equations:8
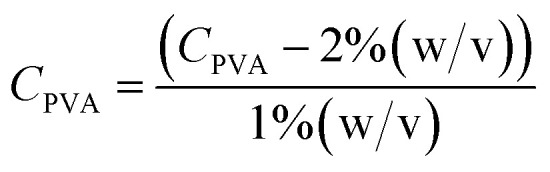
9
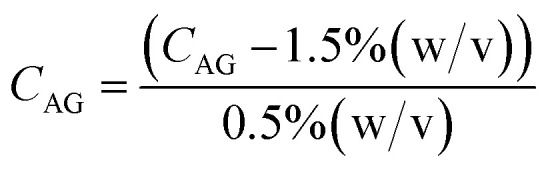
10
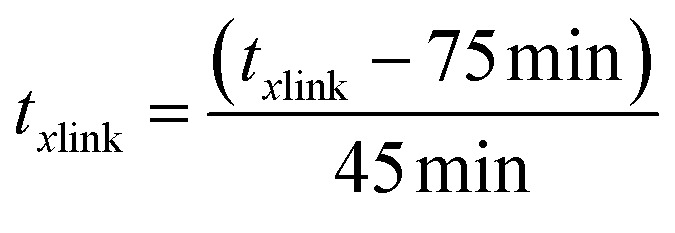


In coded units, the low, medium, and high values correspond to −1, 0, +1, respectively. The number of runs equaled a total of 17 runs with 3 replicates at the design center (Experiment No. 15) to assess the pure error (Table 1). The experimental levels for each variable were determined based on preliminary tests. The rate of transformation of *c*DCE *k*_*c*DCE_, rate of oxygen utilization *k*_O_2__, and compressive modulus *E* after 1 day after immobilization and 30 days after immobilization constituted the dependent variables (responses) in this study. The rate of *c*DCE utilization *k*_*c*DCE_ on day 1 and day 30 was measured for each batch reactor and evaluated with DOE but was excluded from the main text as the primary goal for that data was to show that immobilized cells in all bead types were capable of transforming *c*DCE. Models for predicting *c*DCE can be found in the ESI (Section SI9†).

**Table 1:** Bead formulae for each experiment, generated by a central composite orthogonal design, plus an additional bead formulation (optimal bead). Experimental and predicted data are shown for each experimental condition

No.	*C*_PVA_ [% (w/v)]	*C*_AG_ [% (w/v)]	*t*_*x*link_ [min]	*E*_1_ [kPa]		*E*_30_ [kPa]		Δ*E* [%]		*k*_O_2_,1_ 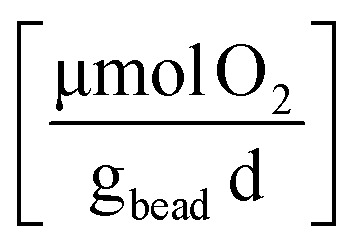		*k*_O_2_,30_ 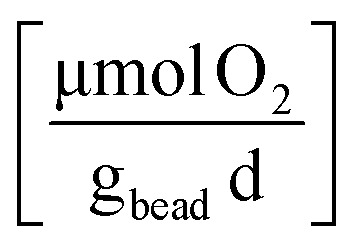	
				Exp.	Pred.	Exp.	Pred.	Exp.	Pred.	Exp.	Pred.	Exp.	Pred.
1	1.0	1.0	30.0	23 ± 0.8	28	5.5 ± 3.3	0.58	76 ± 15	94	30 ± 2.8	28	12 ± 2.1	11
2	3.0	1.0	30.0	60 ± 7.3	56	29 ± 10	29	52 ± 18	58	17 ± 1.6	15	5.1 ± 1.2	4.6
3	1.0	2.0	30.0	75 ± 11	78	13 ± 5.7	21	82 ± 8.1	83	23 ± 2.5	24	6.2 ± 1.3	6.1
4	3.0	2.0	30.0	96 ± 1.5	106	51 ± 6.6	49	47 ± 6.9	48	10 ± 0.9	11	2.8 ± 1.2	3.0
5	1.0	1.0	120.0	70 ± 7.3	55	17 ± 11	21	75 ± 16	69	23 ± 1.6	22	5.7 ± 2.4	5.1
6	3.0	1.0	120.0	71 ± 20	83	30 ± 6.8	27	57 ± 15	60	17 ± 1.5	17	2.2 ± 0.7	1.9
7	1.0	2.0	120.0	103 ± 10	105	45 ± 22	42	56 ± 4.7	59	17 ± 3.2	18	5.9 ± 1.1	6.0
8	3.0	2.0	120.0	127 ± 14	134	26 ± 7.6	47	79 ± 6.4	49	13 ± 2.1	13	5.9 ± 0.8	6.2
9	0.6	1.5	75.0	95 ± 14	31	23 ± 5.4	20	76 ± 6.7	79	18 ± 2.9	28	5.2 ± 0.8	5.6
10	3.4	1.5	75.0	89 ± 5.2	69	40 ± 16	43	SS ± 18	49	24 ± 1.5	16	1.9 ± 0.7	1.4
11	2.0	0.8	75.0	70 ± 7.1	73	8 ± 1.7	11	88 ± 2.8	89	15 ± 1.1	18	5.9 ± 1.5	7.3
12	2.0	2.2	75.0	155 ± 23	40	41 ± 13	39	74 ± 9.5	74	16 ± 1.4	13	7.4 ± 0.7	6.9
13	2.0	1.5	14.1	70 ± 13	31	13 ± 4.0	5.9	81 ± 6.6	77	21 ± 4.5	23	3.5 ± 17	4.4
14	2.0	1.5	135.9	62 ± 16	68	22 ± 3.9	19	64 ± 11	61	21 ± 1.6	21	2.2 ± 0.7	2.6
15	2.0	1.5	75.0	48 ± 8.0	50	8.7 ± 7.8	12	82 ± 16	81	22 ± 1.3	22	4.0 ± 0.3	3.5
Opt	3.2	2.0	110.0	121 ± 10	126	51 ± 11	50	58 ± 9.9	48	7.0 ± 1.0	13	1.2 ± 0.2	5.4

## 3. Results

### 3.1. Cometabolic and metabolic transformation after cell immobilization

**Fig. 2:**
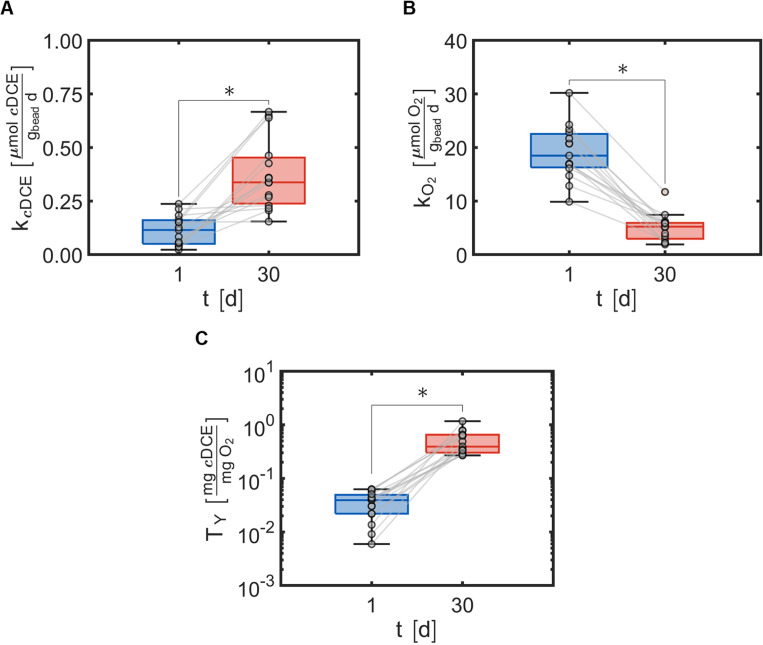
(A) Rate of 1,2-dichloroethylene transformation *k*_*c*DCE_ measured at times, *t* = 1 and *t* = 30 [d], with 15 different bead formulations. (B) Rate of oxygen utilization *k*_O_2__ measured at times, *t* = 1 and *t* = 30 [d], with 15 different bead formulations. (C) Transformation yield *T*_Y_ calculated from rate data at times, *t* = 1 and *t* = 30 [d], with 15 different bead formulations. Data are expressed as box and whisker plots representing the median with the 1st and 3rd quartiles (box) plus the range (whiskers). Circles represent average values of 3 beads per condition, 15 conditions were assessed. The gray lines indicate the change from day 1 to day 30 for each condition. *p < 0.05 by paired *t*-test.

Cometabolic transformation experiments were used to evaluate the capability for ATCC 21198 to perform cometabolism on day 1 and day 30 (Fig. 2A). For all experiments, the rate of *c*DCE transformation increased between day 1 and day 30 indicated by the positive slopes of the lines drawn between *k*_*c*DCE,1_ and *k*_*c*DCE,30_. Values of *k*_*c*DCE,1_ ranged between 0.02 and 0.24 [μmol *c*DCE g_bead_^−1^ d^−1^] for all bead formulations. Values of *k*_*c*DCE,30_ ranged between 0.15 and 0.67 [μmol *c*DCE g_bead_^−1^ d^−1^] for all bead formulations.

Metabolic utilization experiments were used to evaluate the activity of strain ATCC 21198 between day 1 and day 30 (Fig. 2B). For all experiments, the rate of O_2_ utilization decreased between day 1 and day 30 indicated by the negative slopes of the lines drawn between *k*_O_2_,1_ and *k*_O_2_,30_. Values of *k*_O_2_,1_ ranged between 9.9 and 30 [μmol O_2_ g_bead_^−1^ d^−1^] for all bead formulations. Values of *k*_O_2_,30_ ranged between 1.9 and 12 [μmol O_2_ g_bead_^−1^ d^−1^] for all bead formulations.

Transformation yield calculations were used to evaluate the efficiency of the immobilized cells in different formulations of hydrogels with respect to the primary substrate (Fig. 2C). For all experiments, the transformation yield, *T*_Y_, increased between day 1 and day 30 indicated by the positive slopes of the lines drawn between *T*_Y,1_ and *T*_Y,30_. Values of *T*_Y,1_ ranged between 0.0059 and 0.063 [mg *c*DCE/mg O_2_] for all bead formulations. Values of *T*_Y,30_ ranged between 0.27 and 1.2 [mg *c*DCE/mg O_2_] for all bead formulations.

### 3.2. Compressive modulus of hydrogel beads after cell immobilization

**Fig. 3:**
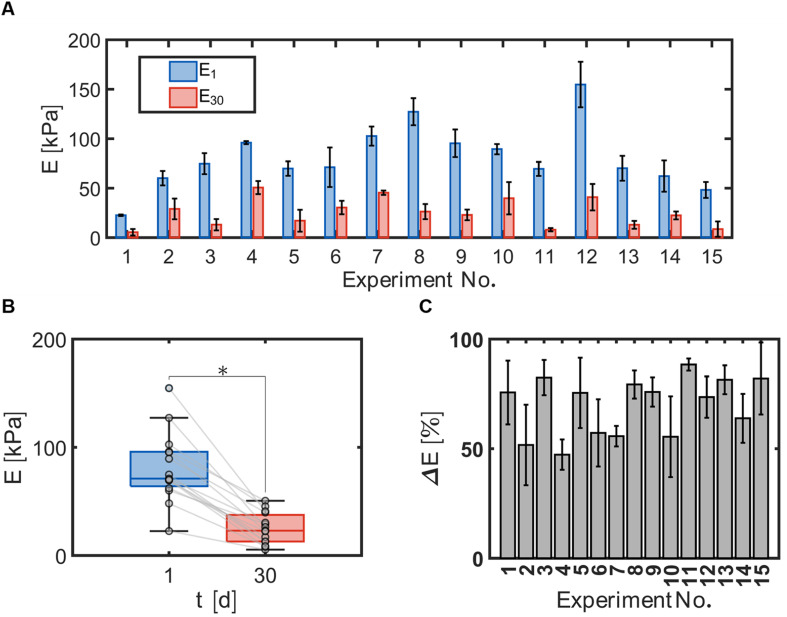
(A) Compressive modulus *E* for each bead formulation at day 1 (*E*_1_) and day 30 *E*_30_. For experiments numbered 1–14 *n* = 3. Experiment 15 is the centrally repeated condition and *n* = 9. All data are expressed as average ± SD. (B) Compressive moduli E measured at times, *t* = 1 and *t* = 30 [d], with 15 different bead formulations. Data are expressed as box and whisker plots representing the median with the 1st and 3rd quartiles (box) plus the range (whiskers). Circles represent average values of 3 beads per condition, 15 conditions were assessed. *p < 0.05 by paired *t*-test. (C) Elastic loss Δ*E* for each bead formulation between day 1 and day 30. For experiments 1–14, data are expressed as average ± SD, *n* = 3. The gray lines indicate the change from day 1 to day 30 for each condition. For experiment 15, data is expressed as average ± SD, *n* = 9.

Hydrogel bead durability was assessed by quantifying the compressive modulus of the hydrogel beads with immobilized cells and TBOS at day 1 and day 30 using the Hertz equation (eqn (3)). The compressive moduli measured for each experiment number (Table 1) at day 1 and day 30 indicated significant differences between bead formulae (Fig. 3A). Comparison of *E*_1_ and *E*_30_ revealed that the compressive modulus decreased for all bead formulae, indicated by the negative slope of the fitted lines between *E*_1_ and *E*_30_ (Fig. 3B). Values for *E*_1_ ranged between 23 and 155 [kPa] for all bead formulations. Values for *E*_30_ ranged between 5.5 and 51 [kPa] for all bead formulations.

Hydrogel bead degradability was characterized by the elastic loss, Δ*E*, or the change in the compressive modulus between day 1 and day 30 with respect to the modulus measured at day 1. The elastic loss demonstrates that beads underwent different degrees of degradation with positive values ranging between 47 and 88 [%] (Fig. 3C).

### 3.3. DOE model fitting and parameters

The central composite design (CCD) is an empirical design that relates the input factors and their combinatorial effects to the responses measured using second-order polynomial response surface equations. We fit second order multivariate regression models for each response and eliminated the terms that were not statistically significant determined from ANOVA tests (*p* < 0.05). Note that lower order insignificant terms must remain in the model if higher order terms or interaction terms involving these terms were deemed significant. The statistical significance of each model was then evaluated with ANOVA tests, with a *p*-value of < 0.05 indicating a significant model and a *p*-value > 0.10 for lack of fit indicating a model with a negligible pure error. For all responses, the models were statistically significant and had negligible pure error (Table 2). The unscaled models obtained for each response variable are as follows:

11 *E*_1_ = 201 + 14*C*_PVA_ − 320*C*_AG_ + 0.3*t*_*x*link_ + 123*C*_AG_^2^

12 *E*_30_ = 41 − 24*C*_PVA_ − 61*C*_AG_ + 0.4*t*_*x*link_ + 11*C*_PVA_^2^ + 27*C*_AG_^2^ − 0.1*C*_PVA_*t*_*x*link_

13 Δ*E* = 94 + 17*C*_PVA_ − 12*C*_AG_ + 0.1*t*_*x*link_ − 9.6*C*_PVA_^2^ − 0.003*t*_*x*link_^2^ + 0.1*C*_PVA_*t*_*x*link_

14 *k*_O_2_,1_ = 14 ± 7.9*C*_PVA_ + 37*C*_AG_ − 0.1*t*_*x*link_ − 14*C*_AG_^2^ + 0.04*C*_PVA_*t*_*x*link_

15 *k*_O_2_,30_ = 41 − 5.4*C*_PVA_ − 33*C*_AG_ − 0.2*t*_*x*link_ + 8.0*C*_AG_^2^ + 1.7*C*_PVA_*C*_AG_ + 0.02*C*_PVA_*t*_*x*link_ + 0.06*C*_AG_*t*_*x*link_

**Table 2:** ANOVA assessment of regression models

Response	*E*_1_	*E*_30_	Δ*E*	*k*_O_2_,1_	*k*_O_2_,30_
***p*-values**					
Model	<0.0001	0.0008	<0.0001	<0.0001	<0.0001
*C*_PVA_	0.0037	0.0009	0.0003	<0.0001	0.0001
*C*_AG_	<0.0001	0.0003	0.0173	0.0161	0.5301
*t*_*x*link_	0.0045	0.0251	0.0148	0.2484	0.0176
*C*_PVA_^2^	—	0.0009	0.0019	—	—
*C*_AG_^2^	0.0001	0.0121	—	0.0061	0.0001
*t*_*x*link_^2^	—	—	0.0133	—	—
*C*_PVA_ *C*_AG_	—		—		0.0147
*C*_PVA_ *t*_*x*link_	—	0.0281	0.0211	0.0386	0.0165
*C*_AG_ *t*_*x*link_			—		—
Lack of fit	0.2079	0.1283	0.6447	0:1697	0.1309

**Validation metrics**					
Total sample size (N)	15	16	15	15	17
Degree of freedom (DF)	10	9	8	9	9
*R* ^2^	0.92	0.92	0.91	0.87	0.94
*R* ^2^-adjusted	0.88	0.86	0.85	0.80	0.90
*Q* ^2^	0.80	0.72	0.66	0.60	0.70

**Fig. 4:**
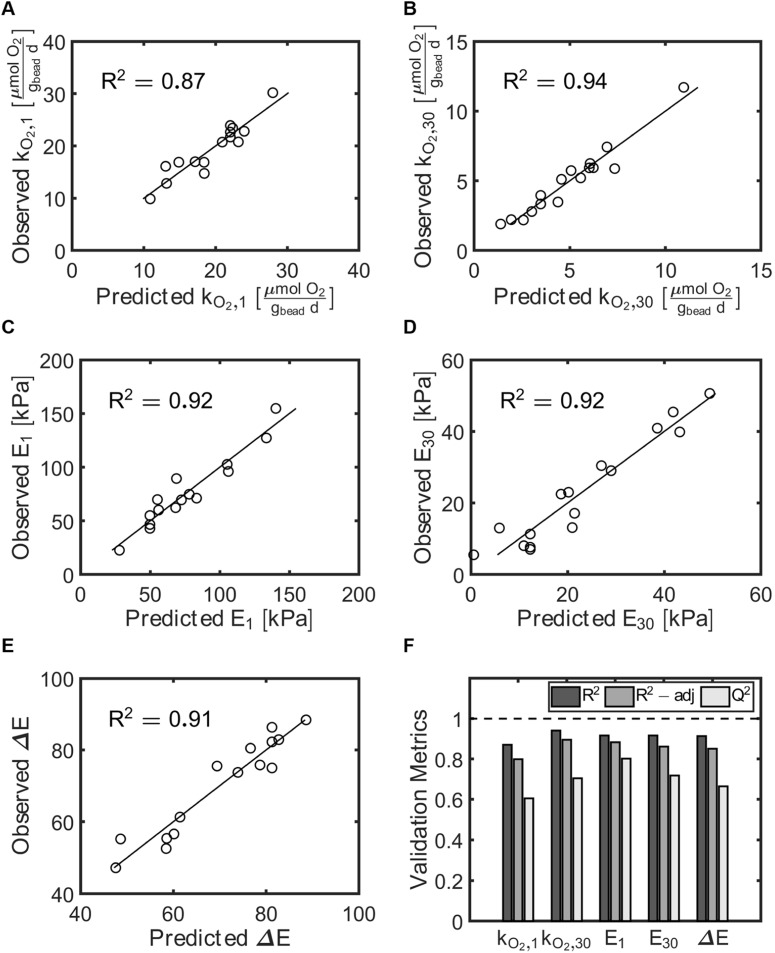
Validation metrics of the response surface models generated from experimental data. (A) Observed *vs.* predicted rates of oxygen utilization at day 1 (*k*_O_2_,1_). (B) Observed *vs.* predicted rates of oxygen utilization at day 30 (*k*_O_2_,30_). (C) Observed *vs.* predicted compressive modulus at day 1 (*E*_1_). (D) Observed *vs.* predicted compressive modulus at day 30 (*E*_30_). (E) Observed *vs.* predicted elastic loss (Δ*E*). (F) *R*^2^, adjusted *R*^2^, and *Q*^2^ for all metrics.

The model performance was further assessed using the coefficients of determination validation metrics: *R*^2^, adjusted *R*^2^, and *Q*^2^ (Table 2, Fig. 4F). *R*^2^ describes the percent of the variation of the response explained by the model for every variable. Adjusted *R*^2^ describes the percent of variation of the response explained by the model only for the variables that affect the response. *Q*^2^ describes the percent of the variation of the response predicted by the model for new data. *R*^2^ and adjusted *R*^2^ values close to 1.0 signal a high correlation between observed and predicted values. *Q*^2^ above 0.50 and the difference between *R*^2^ and *Q*^2^ values lower than 0.30 validates that the model works independently of the specific data used to train the model. We found that all of our models fit the validation metrics criteria, indicating significant correlations between the input variables and the responses. Lastly, the observed response values were compared to those predicted using the second order multivariate regression models (Table 1, Fig. 4A–E). For all responses the observed and predicted values were very close to each other, as indicated by the observed = predicted lines on each of the observed *vs.* predicted plots.

### 3.4. Factor effects and predictions for the rate of oxygen utilization at day 1 (*k*_O_2_,1_) and day 30 (*k*_O_2_,30_)

**Fig. 5:**
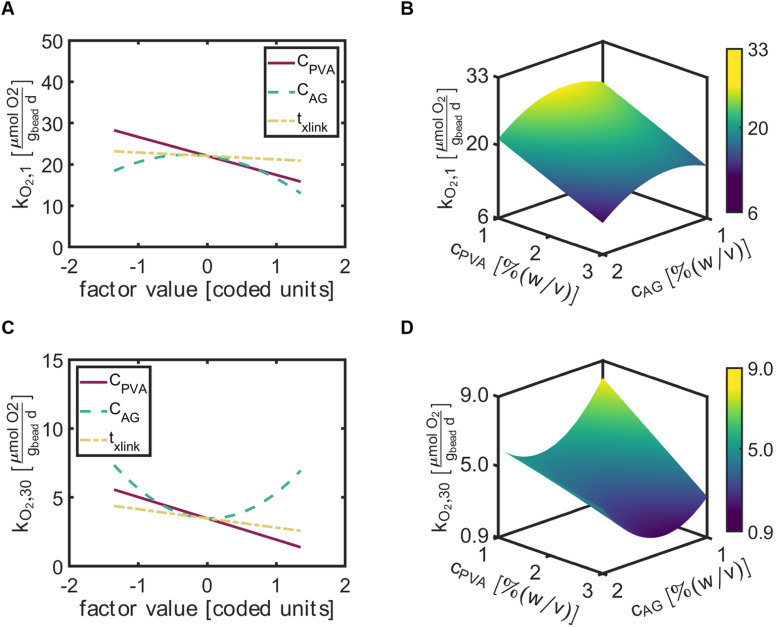
The factor effect plots (left column) include all input variables, *C*_PVA_, *C*_AG_, and *t*_*x*link_ denoted with red (solid), green (dashed), and yellow (dashed-dotted) lines, respectively. 3-D response surface plots (right column) consist of the output response on the *z*-axis predicted for a range (factor value ∈[−1,1]/[coded units]) of *C*_PVA_ and *C*_AG_ on the *x* and *y*-axes, respectively and constant *t*_*x*link_ = 75 [min]. The color bar represents the magnitude of the response from low (purple) to high (yellow). (A) Factor effect plot of *k*_O_2_,1_. (B) 3-D response surface map of *k*_O_2_,1_. (C) Factor effect plot of *k*_O_2_,30_. (D) 3-D response surface map of *k*_O_2_,30_.

The factor effect plot of rate of oxygen utilization at day 1 (*k*_O_2_,1_) indicated *C*_AG_ most significantly influenced *k*_O_2_,1_, with a negative quadratic function centered about *C*_AG_ = 0 [coded units] (Fig. 5A). Thus, relatively high values of *C*_AG_ would result in decreased values of *k*_O_2_,1_. The influence of *C*_PVA_ generated a gentle negative linear slope on the rate of O_2_ utilization. The time for crosslinking had the least impact, with a slight negative slope. The 3D response surface map demonstrates the interactions between *C*_PVA_ and *C*_AG_ on *k*_O_2_,1_ with a constant *t*_*x*link_ = 75 [min] (Fig. 5B). The predicted minimum value of *k*_O_2_,1_ occurred at high values of *C*_PVA_ and *C*_AG_.

At 30 days, the rate of oxygen utilization at day 30 (*k*_O_2_,30_) depended most significantly on *C*_AG_ (Fig. 5C). Interestingly, a positive quadratic curve for the rate of oxygen utilization with respect to *C*_AG_ occurred centered at *C*_AG_ = 0 [coded units] and suggested that both low and high values of *C*_AG_ can provide greater respiration rates. The factor effects *C*_PVA_ and *t*_*x*link_ on *k*_O_2_,30_ revealed linear lines with negative slopes near the same values, indicating that a reduction of the apparent metabolic activity of ATCC 21198 occurred as *C*_PVA_ and *t*_*x*link_ increased. Similar to the response surface map of *k*_O_2_,1_, the predictive plots of *k*_O_2_,30_ suggested that increased values of *C*_PVA_ and *C*_AG_ would result in decreased values of *k*_O_2_,30_ (Fig. 5D). However, the minimum *k*_O_2_,30_ was predicted to occur at *C*_AG_ = 1.6% (w/v) due to the positive parabolic curve exhibited for *k*_O_2_,30_ in response to the factor *C*_AG_.

### 3.5. Factor effects and predictions for the compressive modulus at day 1 and day 30 and the elastic loss

**Fig. 6:**
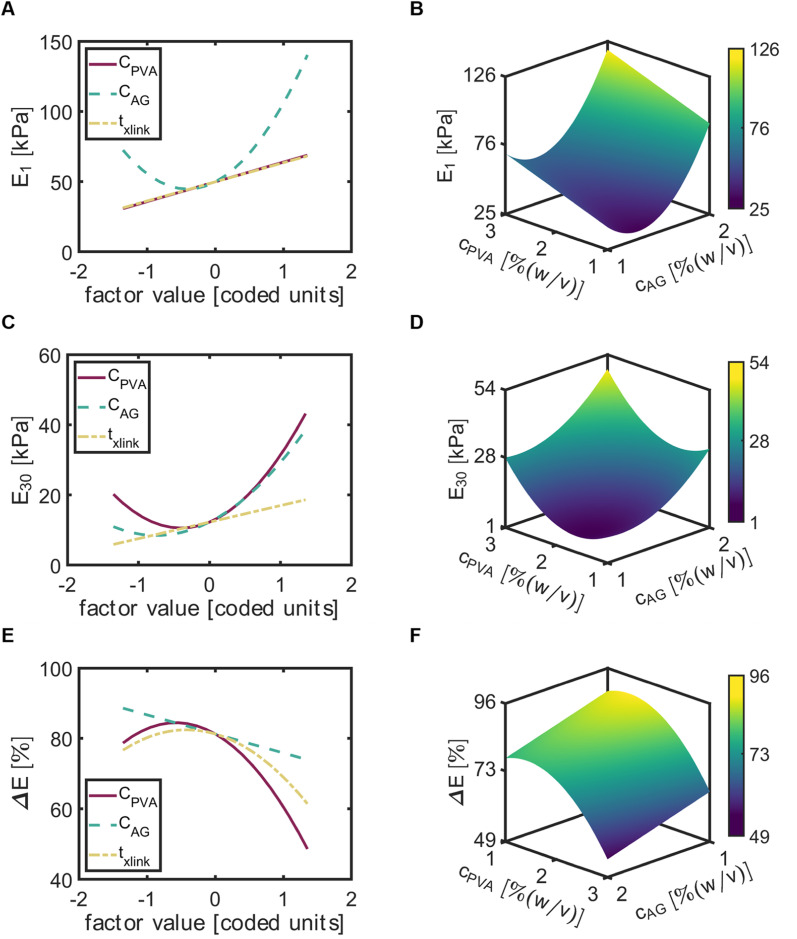
The factor effect plots (left column) include all input variables, *C*_PVA_, *C*_AG_, and *t*_*x*link_ denoted with red (solid), green (dashed), and yellow (dashed-dotted) lines, respectively. 3-D response surface plots (right column) consist of the output response on the *z*-axis predicted for a range (factor value ∈[−1,1]/[coded units]) of *C*_PVA_ and on the *x* and *y*-axes, respectively and constant *t*_*x*link_ = 75 [min]. The color bar represents the magnitude of the response from low (purple) to high (yellow). (A) Factor effect plot of *E*_1_. (B) 3-D response surface map of *E*_1_. (C) Factor effect plot of *E*_30_. (D) 3-D response surface map of *E*_30_. (E) Factor effect plot of Δ*E*. (F) 3-D response surface map of Δ*E*.

For the compressive modulus at day 1 (*E*_1_), *C*_AG_ exhibited a positive quadratic relationship and *t*_*x*link_ exhibited a positive linear relationship. Of the three input variables, *C*_AG_ exhibited the most significant effect (Fig. 6A). The maximum of *E*_1_ with respect to *C*_AG_ occurred at the maximum of AG, while the minimum occurred near the center point (factor value = 0). Both *C*_PVA_ and *t*_*x*link_ factors led to a near similar trend of *E*_1_, with a linear increase in *E*_1_ with increased factors. The response surface map of *E*_1_ indicated that increases in *C*_PVA_ and *C*_AG_ would increase *E*_1_ to a maximum value of 122 [kPa] at *C*_PVA_ = 3% (w/v) and *C*_AG_ = 2% (w/v) (Fig. 6B).

The input variables had similar effects on the compressive modulus at day 30 (*E*_30_). However, in contrast to *E*_1_, *C*_PVA_ exhibited the most significant effect on *E*_30_ and was found to be a quadratic term in the model for *E*_30_ (Fig. 6C). The effects of *C*_AG_ was similar to *C*_PVA_, but with slightly lower values of *E*_30_. The response map of *E*_30_ demonstrated predictions of *E*_30_ would increase with increased values of *C*_PVA_ and *C*_AG_ (Fig. 6D). Here, the maximum value of *E*_30_ was equal to 62.3 [kPa] at *C*_PVA_ = 3% (w/v) and *C*_AG_ = 2% (w/v).

To better decipher the roles of each factor on the decrease of the compressive modulus, we then examined elastic loss (
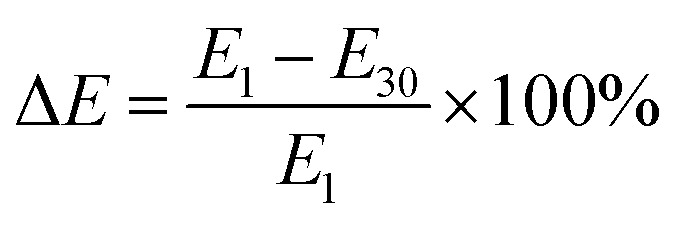
) (Fig. 5E). Interestingly, *t*_*x*link_ exhibited a negative quadratic relationship with Δ*E*, with the maximum Δ*E* observed at a crosslinking time near the center point. A negative quadratic relationship was also observed for *C*_PVA_, with a slight increase in Δ*E* from the minimum to the midpoint yet a steep decline in Δ*E* from the midpoint to the maximum of *C*_PVA_. A negative linear relationship was observed between Δ*E* and *C*_AG_. The response surface map of the Δ*E* revealed that the predicted minimum (Δ*E* = 51%) occurred at *C*_PVA_ = 3% (w/v) and *C*_AG_ = 2% (w/v) (Fig. 6F).

### 3.6. Validation of predictive models to enhance bioremediation potential of ATCC 21198

Our overall engineering objective was to use the second order multivariate regression models to identify the hydrogel formulation that maximized the mechanical strength of the hydrogel beads while simultaneously minimizing the respiration rates of the entrapped microbes. Thus, to validate these models, we generated a hydrogel bead formulation (optimal bead) predicted to fulfill the optimization parameters that was not included in the original CCO design and tested it against model predictions with a confidence at 95% (Table 1). The optimal bead was specified as 3.2% (w/v), 2.0% (w/v), and 110 min for *c*_PVA_, *c*_AG_, and *t*_*x*link_, respectively.

**Fig. 7:**
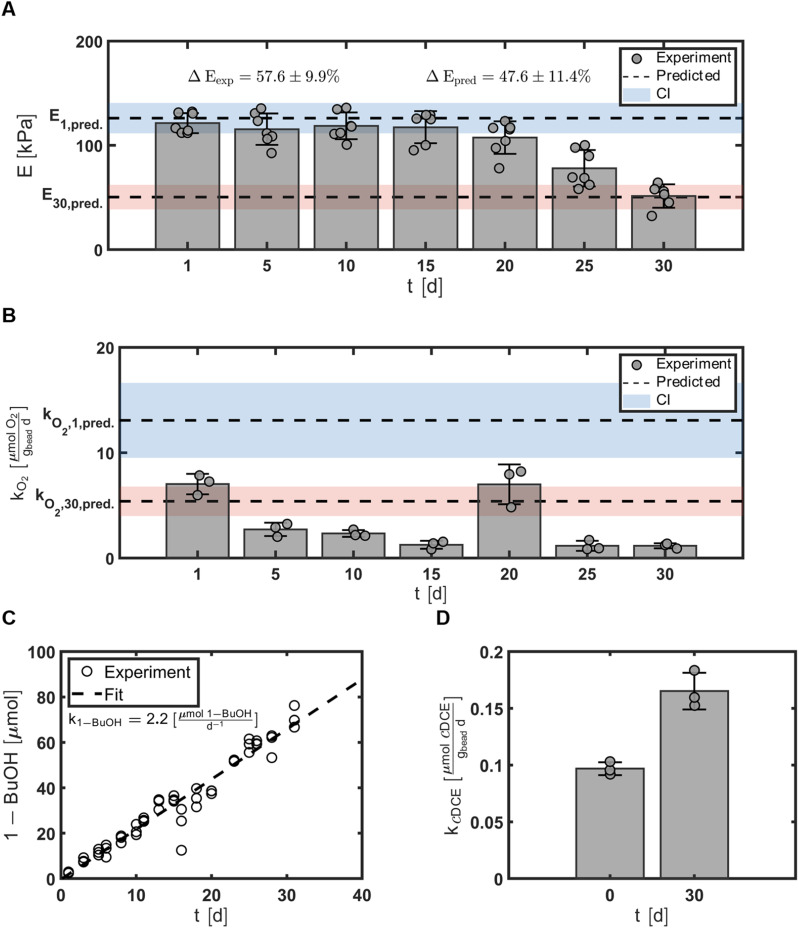
Validation of predictive models. (A) *E* measured for optimal beads. Points represent *E* measured for individual beads from batch reactors and bars represent the average ± SD, *n* = 7. The experimentally determined elastic loss, Δ*E*_exp_, and predicted elastic loss, Δ*E*_pred_, are shown on the upper left and right of the figure, respectively. (B) Rate of oxygen utilization *k*_O_2__ measured from batch reactors with optimal beads. Points represent *k*_O_2__ measured for each batch reactor and bars represent the average ± SD, *n* = 3. For figures A and B, predicted values for day 1 and day 30 are shown with dotted lines with confidence intervals colored with blue and red, respectively. (C) Abiotic hydrolysis data. Points represent the measured 1-BuOH in each batch bottle and the dotted line represents the zero-order fit, used to determine the rate of 1-BuOH. (D) *k*_cDCE_ measured at times, *t* = 1 and *t* = 30 [d], for batch reactors with optimal beads. Data are expressed as average ± SD, *n* = 3.

The mechanical strength of the hydrogel beads was evaluated by measuring the compressive moduli, *E*, every five days for 30 days, *t* ∈ {1, 5, 10, 15, 20, 25, 30}\[d], that resulted in average values of *E* = {121, 115, 119, 117, 107, 78, 51}\[kPa], respectively (Fig. 7A). *E*_1_ was 121 ± 10 and 126 kPa for the measured and predicted values, respectively. *E*_30_ was 51 ± 11 and 50 kPa for the measured and predicted values, respectively. The optimal bead experienced an elastic loss, Δ*E*, of 58 ± 9.9% compared to the predicted value at of 48%. For moduli data, the experimental mean or standard deviation overlapped the prediction interval with confidence at 95%.

The metabolic activity of the entrapped microbes was evaluated by measuring the oxygen consumption rates every five days for 30 days, *t* ∈ {1, 5, 10, 15, 20, 25, 30}\[d], that resulted in average values of *k*_O_2__ = {7.0, 2.7, 2.3, 1.3, 7.0, 1.2, 1.2}\[
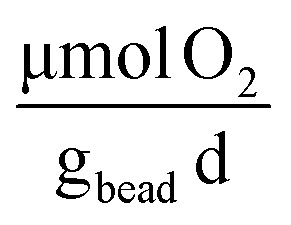
], respectively (Fig. 7B). On day 1, rates of oxygen utilization *k*_O_2_,1_ measured with the optimal bead was 7.0 ± 1.0, whereas the predicted value was 13 (Fig. 7A). The rate of oxygen utilization at day 30, *k*_O_2_,30_, was measured at 1.2 ± 0.2 
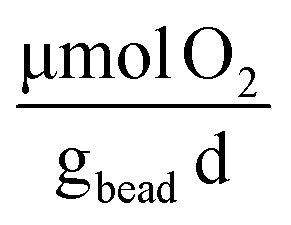
 compared to the predicted value of 5.4 
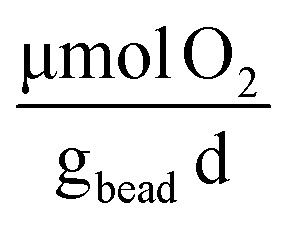
 (Fig. 7B).

### 3.7. Rate of abiotic hydrolysis of TBOS for optimized beads

Abiotic hydrolysis tests were performed to evaluate the behavior of the slow-release compound without cells present. The abiotic hydrolysis of TBOS exhibits a zero-order rate, with the amount of 1-butanol increasing linearly over time, and serves as a proxy for how 1-butanol hydrolyzes within the bead in the absence of microbial utilization (Fig. 7C). This data can elucidate how well the rate of oxygen utilization data can predict hydrolysis rates based on a stoichiometric balance between 1-butanol and oxygen. For the optimal bead, we observed an abiotic hydrolysis rate at 1.1 
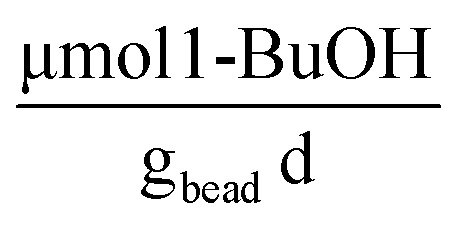
.

### 3.8. Rate of transformation of cDCE for optimized beads

Cometabolic activity was evaluated by measuring the transformation of *c*DCE at times, *t* = 1 and *t* = 30 [d] (Fig. 7D). The rate of *c*DCE was measured at 0.10 ± 0.01 
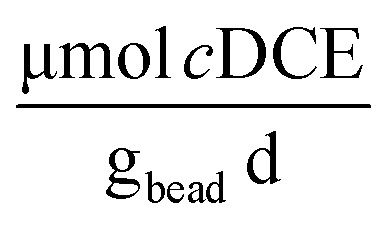
 at day 1, and increased to 0.17 ± 0.02 
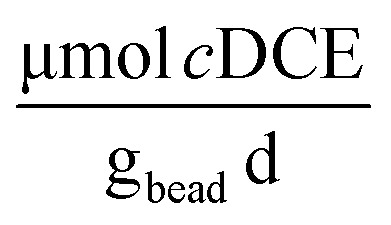
 at day 30.

### 3.9. Transformation yields for optimized beads

The transformation yield *T*_Y_ was calculated at days 1 and 30 to evaluate the efficiency of the immobilized cells in the optimal beads. *T*_Y_ was 0.084 ± 0.016 and 0.86 ± 0.21 
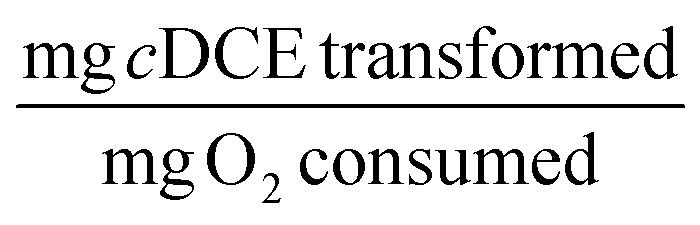
 at day 1 and 30, respectively.

## 4. Discussion

Measurements of the transformation of *c*DCE between day 1 and day 30 demonstrated the capability for ATCC 21198 to perform cometabolism when immobilized in different compositions of PVA-AG hydrogel beads. Statistical modeling with DOE suggested that the compressive modulus (*E*), the elastic loss (Δ*E*) of the hydrogel beads, and the oxygen utilization rates of ATCC 21198 (*k*_O_2__), could be modeled as functions of all three of the input variables that composed the hydrogel formulation, *C*_PVA_, *C*_AG_, and *t*_*x*link_. The hydrogel composition directly affected *E* at day 1 and day 30, such that the maximum value of *E* would occur at the maximum levels of *C*_PVA_, *C*_AG_, and *t*_*x*link_. Additionally, the models predicted that the minimum values of Δ*E* and *k*_O_2__ at day 1 and day 30 would result from the maximum values of all three input variables. The validation of the optimal hydrogel formulation demonstrated the capability for the second order multivariate regression models to capture the material properties based on compressive moduli data after initially making the beads and 30 days after an incubation period with cells. The biological response used throughout this study, *k*_O_2__, needs further investigation as the models could not predict *k*_O_2__ at day 1 or day 30 observed in the optimal batch study. Still, these models are a first attempt to empirically optimize the material properties and biological cues based on hydrogel formulae for this *in situ* bioremediation strategy.

Transformation of *c*DCE measurements ensured that cometabolic transformations were possible across all bead formulations. Further, rates of *c*DCE transformation (*k*_*c*DCE_) increased during the 30 day incubation across all bead formulations. Murnane *et al.* reported minimal induction of the monooxygenase enzyme used to transform *c*DCE with a 1-butanol substrate for ATCC 21198.^38^ We postulate that *k*_*c*DCE_ increased over a 30 day period due to an increase in ATCC 21198 population between day 1 and day 30 (Fig. 2A); yet recognize that the monooxygenase enzyme may be stimulated in the presence of *c*DCE.^20^ Our previous works have demonstrated that ATCC 21198 can also cometabolize other CAHs and co-contaminants, such as 1,1,1-trichloroethane, and 1,4-dioxane.^12,20^ We intended to use *c*DCE as a surrogate for cometabolism, and due to the capability for ATCC 21198 to transform *c*DCE throughout all bead formulations, we have demonstrated that this immobilization method could be extended to treat other CAHs, and 1,4-dioxane in column studies.^39,40^

The rates of oxygen (O_2_) utilization, *k*_O_2__, were used to estimate the metabolic activity that corresponded with the utilization of 1-butanol (1-BuOH), the product of hydrolyzed TBOS (Fig. 2B). Unlike *c*DCE transformation data, *k*_O_2__ decreased over a 30 day period. Due to the environmental changes the cell undergoes (from growth medium to hydrogel precursor solution to crosslinking bath and finally back to medium), the increased oxygen rate at day 1 could occur from environmental stress. The crosslinking bath is naturally acidic (pH ∼4.5) due to the boric acid that crosslinks PVA, and could damage cells during the crosslinking process. Thus, the rate of oxygen utilization on day 1 may be partially linked to cell repair. Second, the low pH of the crosslinker bath could rapidly hydrolyze TBOS and flood the hydrogel with plenty of 1-BuOH to initiate a high rate of oxygen utilization. The abiotic hydrolysis tests performed for the optimal bead trial suggests that the latter is less likely as the amount of 1-BuOH present in batch bottles at day 1 is approximately zero; however, it should be noted that the TBOS used for optimal beads was of higher purity (98% purity) compared to the CCO experiments (90% purity) and the residual could contain pure 1-BuOH. Indeed, a spike in 1-BuOH at day 1 would promote greater respiration rates and could lend reasoning behind missed predictions for the optimal bead trials. This information heavily warrants that the amount of substrate should be accounted for in future models.

While uncertainties in the behavior of *k*_O_2__ exist, we attempted second order multivariate modeling for *k*_O_2_,1_ and *k*_O_2_,30_ to identify trends, and found these dependent variables significantly depended on all three factors as well as interaction terms. We found that in general, increasing values of *C*_PVA_ would result in decreased responses of *k*_O_2_,1_ (Fig. 5A and B) and *k*_O_2_,30_ (Fig. 5C and D). Kumar *et al.* describe PVA as an oxygen barrier, which may explain the factor effect behavior of *C*_PVA_ on *k*_O_2__.^41^ Further, the significant decrease of *k*_O_2_,1_ could occur due to an increase in the total polymer volume fraction.^42^ The polymer volume fraction can be thought as the amount of volume the polymer takes up in the gel in comparison to free volume for diffusion. Higher amounts of polymer content could cause tighter gel networks and decrease cells capability to proliferate and reduce the total respiration rate. A negative quadratic slope was predicted for *k*_O_2_,1_ with respect to *C*_AG_ and contributes to the lowest *k*_O_2_,1_ value at a maximized factor value *C*_AG_ = 1.4 [coded units] (Fig. 5B). Similar to the reasoning for the *k*_O_2__ values in respect to *C*_PVA_, greater polymer content could lead to lower oxygen utilization rates due to the incapability for cells to proliferate. *k*_O_2_,30_ was modeled as a positive quadratic curve with respect to *C*_AG_, suggesting that high concentrations of alginate would indeed increase *k*_O_2_,30_ (Fig. 5D). Differences in cell density could tend to drive higher oxygen rates, and due to characteristic biocompatibility that alginate possesses, beads with higher amounts of alginate could yield a greater population of cells at day 30.

The crosslinking time (*t*_*x*link_) exhibited a significant negative linear effect on both *k*_O_2_,1_ and *k*_O_2_,30_ (Fig. 5A–D). This may be due to the fact that during crosslinking the cells are exposed to boric acid and the longer cells interact with the acidic crosslinker, the more cells could undergo cell death.^43^ As more cells perish, the respiration rates would be lower for hydrogels with longer crosslinking times compared to shorter time, assuming equal amounts of cells are immobilized. An additional consideration, and more closely related to total polymer content, is that as *t*_*x*link_ increases, the gels proceed toward equilibrium and more available crosslink sites are inhabited by ions. A gel with more crosslinked sites would have, on average, smaller pore sizes, and lower effective diffusivity that would limit cell proliferation.^44,45^

The statistical models for *k*_O_2_,1_ and *k*_O_2_,30_ could suggest trends between the factors related to the bead formulae. However, these models must be used with caution when predicting values of oxygen rates. Specifically, we found that the optimal bead validation tests resulted in *k*_O_2__ values that did not overlap with predicted values. An analysis of the rates of oxygen utilization for an alginate only hydrogel positive control (*C*_PVA_ = 0 [% (w/v)], *C*_AG_ = 1.5 [% (w/v)], and *t*_*x*link_ = 75 [min]) demonstrates the differences in respiration rates with different batches of cells (Section ESI10: Fig. S5†). This suggests that the differences are not necessarily due to the bead formula. Instead, the differences could occur from differences in the number of cells per bead. The cell inoculum concentration is set in the bead precursor solution, and cells may not be evenly distributed amongst the beads. Secondly, the quantity of live *versus* dead cells is not known. Without methods to determine live *versus* dead cells before cells are added to the bead precursor solution or after the cells are inoculated in beads, we cannot account for differences in the quantity of live cells present for each batch of beads. Potential live/dead staining techniques used to determine cell counts and live/dead ratios in beads could be available to use for our *Rhodococcus* strain similar to the work of Veeranagouda *et al.*;^46^ however, that methodology has yet to be determined for our bacteria strain in a hydrogel system. Note that due to the unknown quantity of cells in the beads means that we cannot compare either *k*_*c*DCE_ or *k*_O_2__ between this study and others.

Stoichiometric analysis of the O_2_ consumption compared to the abiotic rates of hydrolysis from TBOS forces us to reconsider the interactions between the cells and the slow-release compound. We consider the stoichiometric balance: 6 μmol of O_2_ is required to oxidize 1 μmol 1-BuOH to carbon dioxide and water. Altogether, the total amount of oxygen consumed for the optimal batch beads and the total amount of 1-BuOH liberated from abiotic beads over the 30 days was 215 μmol O_2_ and 66 μmol 1-BuOH. The ratio between oxygen consumed and 1-BuOH produced equals 3.3 
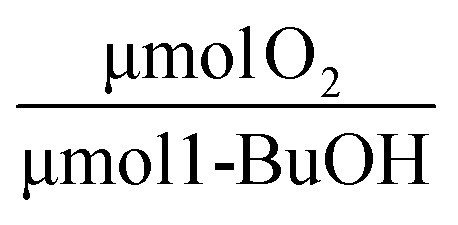
, 1.8 times less than stoichiometrically expected. In our previous works, we found that cells in our gellan gum matrix coupled with TBOS consumed nearly twice the expected oxygen.^20^ The discrepancy between this work and our previous work is likely a consequence of the use of 1-BuOH for cell synthesis.

To consider the efficiency of immobilized cells in different hydrogel formulations with respect to the primary substrate, 1-butanol, we calculated *T*_Y_ at days 1 and 30. For the optimal beads, *T*_Y_ is equal to 0.084 ± 0.016 and 0.86 ± 0.21 
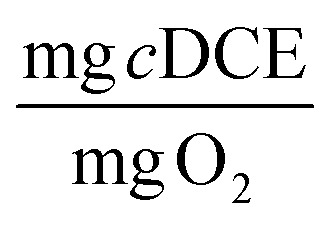
 at days 1 and 30. In our previous work with ATCC 21198 immobilized in gellan gum, we obtained *T*_Y_ values of 0.012 ± 0.005 and 0.015 ± 0.02 
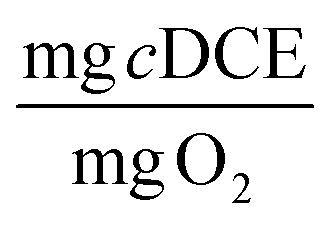
 at days 2 and 32.^20^ While the values for *T*_Y_ found in our previous work were an order of magnitude less than the optimal beads studied in this work, the reactors in our previous work were subjected to injections of 1,1,1-trichloroethane, 1,4-D, and *c*DCE at concentrations ranging approximately between 250 and 1000 μg L^−1^, which were also transformed to a great extent. Therefore, we would expect the values of *T*_Y_ to be greater for the beads tested in this study. In this study, we saw significant increases between the values of *T*_Y_ measured at day 1 and 30, whereas Rasmussen *et al.* did not see a significant increase in *T*_Y_. Therefore, we suspect that more growth was observed in this study and is likely due to a difference in the structure of PVA-AG beads compared to gellan gum hydrogels beads.^47^ Based on the assumption that the consumed oxygen serves as the surrogate for 1-butanol, we use the value for the ratio between oxygen consumed and 1-BuOH produced to calculate *T*_Y_ based on 1-BuOH. This results in *T*_Y_ equal 0.06 ± 0.011 and 0.61 ± 0.15 
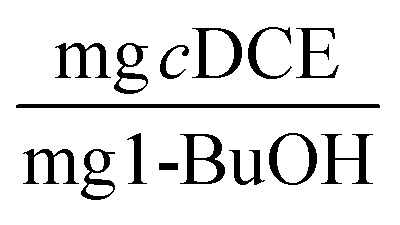
. Tejasen studied an aerobic mixed culture grown on TBOS and observed a *T*_Y_ of 0.24 
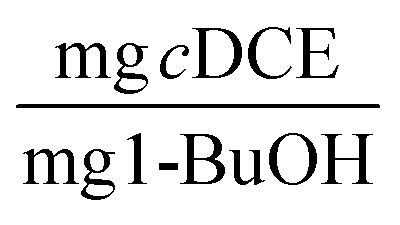
.^48^ The values for *T*_Y_ likely differ due to the differences of the microorganisms between the different studies.

In regard to the material properties of the hydrogels, depletion of hydrogel bead mechanical properties was observed. Significant differences were found between the compressive modulus, *E*, at day 1 and day 30 (Fig. 3B) for all bead types. The decrease in the compressive modulus, *E*, and the increase in the elastic loss, Δ*E*, over time likely occurred due to a loss of crosslinking between polymer chains. Several possibilities can describe the potential loss of crosslinking: (1) calcium ions could transfer from alginate linkages to phosphate ions in solution to form calcium phosphate.^49^ The MSM used in the incubation batch reactors contains excess phosphate ions that could reduce the crosslinks between calcium and alginate polymers. (2) Crosslinks between PVA and boric acid could be reduced as they are labile bonds. Casassa *et al.* discuss the dynamic equilibrium of a PVA hydrogel due to the labile hydrogen bonds that must form between PVA and boric acid, thus these crosslinks could have been reduced due to long periods of shaking.^50^ (3) Cell proliferation could reduce crosslinking. With enough pressure within the hydrogel matrix due to cell growth promoted with the use of TBOS, the increase of cell population could have broken the crosslinks in the polymer network (reduced the crosslink density) and further reduced the compressive modulus. Comparison between the CCO center point experiment (Experiment No 15) and an abiotic experiment with beads with the same formulation demonstrates that hydrogels with cells that can proliferate will undergo greater elastic loss (Section 11: Fig. S6†). The data observed for our optimal bead formulation demonstrates the change in *E* over time in and suggests good stability of hydrogels for at least 20 days (Fig. 7A). While there is a decline in the compressive moduli over time, additional studies performed outside the scope of this work demonstrated that these hydrogel beads would retain their shape and provide the capability for 21 198 to transform contaminants for over one year in column studies.^39,40^

The accurate models used to predict the compressive moduli could be used to describe the effects of the input factors on *E*_1_, *E*_30_ and Δ*E*. In general, the models suggest that increases in *t*_*x*link_, *C*_PVA_, and *C*_AG_ result in increased compressive moduli for both days (Fig. 6A–D). This is in line with many other studies that have demonstrated that increasing polymer concentration and crosslinking time increases compressive moduli across a wide range of hydrogels.^51,52^ Evaluation of factors on the elastic loss demonstrated how hydrogel degradation occurred over the 30 day incubation period. We observed that beads with greater mechanical strength, obtained from high polymer content and crosslinking time, degraded less compared to the other formulae over the 30 day incubation period. Additionally, the squared term *C*_AG_^2^ is significant in *E*_1_, *E*_30_ and Δ*E*, and *C*_PVA_^2^ is significant in *E*_30_ and Δ*E*. These squared terms are expected as hydrogel systems have been shown to exhibit a power law behavior for the compressive modulus.^53^ However, the response surface map of Δ*E* demonstrates that both *C*_PVA_ and *C*_AG_ at high concentrations alone do not support gel structure (Fig. 6E–F). Only when *C*_PVA_ and *C*_AG_ values appear at the maximums together does the response of elastic loss significantly decreases. This behavior indicates synergy between PVA and AG blends that reduces the capability for chemical crosslinks to dissociate in aqueous solutions. As mentioned above, bead degradation could occur through abiotic processes.^49,50^ In the case of PVA polymers, due to the continual breaking and reforming of chains, the structure can be supported by alginate crosslinks where PVA could reform in the gel without losing polymer content. Overall, the synergistic interactions between PVA and AG promote a more durable hydrogel.^54–56^

Here we discuss the interaction terms found in eqn (11)–(15). The interaction term *C*_PVA_*t*_*x*link_ was found to be significant for *k*_O_2_,1_, *k*_O_2_,30_, *E*_30_, and Δ*E*. We find that at high concentrations of *C*_PVA_, increases in *t*_*x*link_ increases *k*_O_2_,1_, *k*_O_2_,30_ and Δ*E*, but decreases *E*_30_. At low concentrations of *C*_PVA_, increases in *t*_*x*link_ results in a decrease in *k*_O_2_,1_, *k*_O_2_,30_ and Δ*E*, but increases *E*_30_. Liao *et al.* evaluated the morphology of PVA beads crosslinked with boric acid and observed an internal porous structure with many irregular pores, yet found no obvious pores on the surface of the beads.^57^ Their explanation is that gelation is quick at the surface which reduces the permeability of the individual beads. An increase in *t*_*x*link_ increases the time for ions to diffuse through the highly crosslinked surface and generate the internal porous structure. At high *C*_PVA_, the viscosity of the pre-gel solution is higher which reduces diffusion. Further, higher concentrations of PVA lead to more entanglements available to crosslink, and more ions will be taken up as they diffuse into the bead. Thus, at high *C*_PVA_, increasing *t*_*x*link_ allows more ions to populate the dense number of crosslink sites and create an internal structure further inside the bead. This in turn allows for better transport of 1-butanol, which increases the respiration rate, and decreases the compressive modulus similar to a sponge. At low *C*_PVA_, increases in *t*_*x*link_ promotes further crosslinking to create a denser network. Since diffusion is not as affected at low *C*_PVA_*vs.* high *C*_PVA_, we expect that the pores get smaller at *t*_*x*link_ increases due to more crosslinks. As pores get smaller, respiration rates decrease, and the compressive modulus increases. Interestingly, the model for *k*_O_2_,30_ also included the interactions *C*_PVA_*C*_AG_ and *C*_AG_*t*_*x*link_. *C*_PVA_*C*_AG_ was found to have a positive interaction on *k*_O_2_,30_, which could be due to the synergy described before. *C*_AG_*t*_*x*link_ was also positive, which could be due to more time for calcium ions to saturate the gel and provide a better structure for cells over the 30 day incubation period.

A critical finding established in this work elucidates the capability to predict mechanical properties of a hydrogel bead with immobilized 21 198 and slow-release compounds based on the hydrogel composition. We demonstrated that the optimal bead formula exhibited a relatively high compressive modulus, low elastic loss, and low rate of oxygen utilization for both 1 and 30 days. The optimal bead formula determined from this study would promote longer periods of bioremediation and reduce the frequency that beads would need to be replenished in permeable reactive bio-barriers. The use of statistical designs and response surface plots can help investigate the interactions between tunable variables and the cell response that occurs in cell–hydrogel interactions;^47^ however, we have shown that respiration data cannot be described by hydrogel formulations alone. Of course, this finding is due to the interactions between cells and hydrogels that obscure our understanding of the mechanisms that exist, which suggests that models need more and more experimental and computational validation.^47^

Lastly, batch reactors with mineral salt medium (MSM) may not capture the effects that groundwater composition and conductivity can impose on the beads. MSM provides excess nutrients to the microorganisms that promote growth but can potentially reduce the number of crosslinks in hydrogels due to the concentration of phosphate ions that exist in the MSM (760 mg L^−1^ PO_4_^−3^). Groundwater typically possesses significantly less phosphate than the MSM (<10 mg L^−1^ PO_4_^−3^).^58^ With lower amounts of nutrients for cells and significantly less phosphate, we expect cell growth would be slower and the reduction of crosslink sites would occur less. Thus, we expect cells would exhibit lower rates of metabolic activity and that beads would experience less elastic loss over time when compared to the values observed throughout this study.

## 5. Conclusions

We have demonstrated the capability of poly(vinyl)-alcohol – alginate beads for the immobilization of ATCC 21198 with a slow-release compound, tetrabutoxysilane. With the use of a Design of Experiments approach, we were able to elucidate factors of the hydrogel formulation that provide high mechanical strength but were unable to accurately predict oxygen utilization rates. While more in-depth measurements on the interactions between the slow-release compound, oxygen rates, and hydrogel formula are needed, these beads would provide cells with a stable, durable microenvironment and a constant carbon source. These beads have already been applied in long-term column studies and could be applied as an *in situ* bioremediation technique to reduce CAHs, and 1,4-dioxane in groundwater aquifers.^39,40^

Future studies will consist of scaling-up the production of hydrogel beads that control the size and output using a coaxial air and piezoelectric ring set-up, as well as characterizing the hydrogel structure with molecular and structural characterization techniques. There is still a need to determine the number of cells that develop within the beads over time and how the hydrogel structure responds to changes in cell density. Additionally, future studies will be conducted with actual groundwater samples to demonstrate the durability and bioremediation capability of immobilized cells and support the feasibility of this approach.

## Data availability

All data are available on the https://github.com/fogg-lab/.

## Conflicts of interest

The authors have no conflicts to declare.

## Supplementary Material

SU-002-D3SU00409K-s001
