# Supplementary material for: The optimization of poly(vinyl)-alcohol-alginate beads with a slow-release compound for the aerobic cometabolism of chlorinated aliphatic hydrocarbons
Source: RSC Sustain. 2024 Mar 14;2(4):1101–17. doi: 10.1039/d3su00409k (PMC10993105; doi:10.1039/d3su00409k)
Supplement: SU-002-D3SU00409K-s001 [file SU-002-D3SU00409K-s001.pdf]

Electronic Supplementary Material (ESI) for RSC Sustainability.
This journal is © The Royal Society of Chemistry 2024

Supplemental Information

SI1. *Mineral Salt Medium*

Mineral salt medium (MSM) was prepared as we described in Rolston et al.(1) MSM consists of two solutions, namely Solution 1 and Solution 2. Unless otherwise noted, ultra-pure water was obtained using a PURELAB flex 1 – ELGA LabWater water purification system (Veolia Water, Paris, France). Solution 1 consists of 2% (w/v) ammonium chloride (Thermo Fisher Scientific Inc), 0.075% (w/v) magnesium chloride (Mallinckrodt Baker, Inc., Phillipsburg, New Jersey, United States), 0.1% (w/v) ammonium sulfate (VWR International, Radnor, Pennsylvania, United States), and 0.2% (v/v) trace elements dissolved into ultra-pure water (**Table S1**). Before the trace elements are added to Solution 1, they are dissolved into ultra-pure water and neutralized to a pH of 7 using potassium hydroxide (Thermo Fisher Scientific Inc, Waltham, Massachusetts, United States. Solution 1.

Table S1 1X MSM solution compounds

Solution	Compound	Concentration [% (w/v)]	Final Concentration in 1X MSM [% (w/v)]
1	Ammonium chloride (Thermo Fisher Scientific Inc, Waltham, Massachusetts, United States)	2.0	0.20
	Magnesium chloride (Mallinckrodt Baker)	7.5E-02	7.5E-03
	Ammonium sulfate (VWR International)	0.10	1.0E-02
	Ethylenediaminetetraacetic acid (VWR International)	1.0E-02	1.0E-03
	Zinc sulfate heptahydrate (Merck KGaA, Darmstadt, Germany)	4.4E-03	4.4E-04
	Manganese dichloride tetrahydrate (VWR International)	1.0E-03	1.0E-04
	Ferrous sulfate heptahydrate (Mallinckrodt Baker)	1.0E-03	1.0E-04
	Calcium chloride (Merck KGaA)	9.0E-04	9.0E-05
	Cobalt chloride hexahydrate (Sigma-Aldrich, St. Louis, Missouri, United States)	3.4E-04	3.4E-05
	Copper sulfate pentahydrate (Thermo Fisher Scientific Inc)	3.0E-04	3.0E-05
	Ammonium molybdate tetrahydrate (Beantown Chemical Corporation, Hudson, New Hampshire)	2.2E-04	2.2E-05
2	Dipotassium hydrogen phosphate (Thermo Fisher Scientific Inc)	15.5	0.155
	Monosodium phosphate (Sigma-Aldrich)	8.50	8.50E-02

Solution 2 consists of 15.5% (w/v) dipotassium hydrogen phosphate (Thermo Fisher Scientific Inc, Waltham, Massachusetts, United States) and 8.5% (w/v) monosodium phosphate (Sigma-Aldrich, St. Louis, Missouri, United States) dissolved into ultra-pure water (**Table 1**). Solution 1 and solution 2 are autoclaved separately to ensure reactions between compounds occur. Finally, Solution 1 and Solution 2 are diluted with deionized water at a 10:1:100 ratio to form a 1X MSM solution.

SI2. ***ATCC 21198 Cell culture minimal plates***

ATCC 21198 was maintained on minimal medium plates in a sealed lock tight jar with 45 mL of isobutane (Gas Innovations, La Porte, Texas, United States) at 30 ˚C. Minimal medium plates were prepared as we previously described in Rolston et al.(1) A solution of 1.7% (w/v) Difco 247940 agar (Thermo Fisher Scientific Inc) was dissolved in ultra-pure water then autoclaved. Once cooled (~< 30 ˚C), MSM was added to the agar solution and the solution was poured into polystyrene disposable sterile petri dishes (VWR International) to form gels. The culture is validated by streaking heterotrophic growth plates made with premixed Difco 247940 agar (Thermo Fisher Scientific Inc, Waltham, Massachusetts, United States) or with 3, 10, and 15 % (w/v) of tryptic soy, glucose, and agar, respectively, in ultra-pure water (1).

SI3. ***ATCC 21198 liquid culturing***

Liquid culture growth reactors were used to grow ATCC 21198 as we previously described in Rolsten et al.(1) The growth reactors consisted of 720 mL Wheaton bottles (DWK Life Sciences Wheaton, Stoke-on-Trent, United Kingdom) sealed with screw-on caps fitted with gray butyl rubber septa (DWK Life Sciences Wheaton) filled with 300 mL of phosphate-buffered 1X MSM at a pH of 7.0 and 420 mL of air headspace. ATCC 21198 were inoculated by using an inoculating loop (VWR International) to scrape minimal medium plates (see above) and place an inoculum of ATCC 21198 into our growth reactor MSM. The bottles were then sealed, injected with 50mL of isobutane (Gas innovations, La Porte, Texas, United States) into the headspace, and placed the growth reactors on a New Brunswick Scientific G10 Gyratory

shaker table (Eppendorf, Hamburg, Germany) at 200 RPM at 30 ˚C to incubate. Cell harvest ensued by harvesting ATCC 21198 in the late exponential growth phase by centrifugation with a Beckman J2-MI Centrifuge (Beckman Coulter Inc., Brea, California, United States) equipped with a JA-14.50 Fixed-Angle Rotor (Beckman Coulter Inc.) at 8000 RPM for 8 min in 300 mL Nalgene bottles (Thermo Fisher Scientific). Following initial centrifugation, all of the cell pellets from individual bottles were combined into 50 mM monosodium phosphate (Sigma-Aldrich) at a pH of 7 and repeated centrifugation and decanting of the supernatant.

An aliquot of suspended cells (v_l) was pipetted using a Finnpipette Digital 40-200 µL pipette (Thermo Fisher Scientific) with 200 µL pipet tips (VWR International) onto 0.2 µm Membrane filter paper (Whatman, Maidstone, United Kingdom) with initial weight (m_i) under vacuum to remove excess liquid. To remove the remaining liquid, we subjected the filter paper to a VWR Oven F Air 6.3CF oven (VWR International) at a temperature of 105 ˚C for 20 min to provide the necessary energy to dry the sample.

A measurement of the total suspended solids (TSS) provided the concentration of suspended cells as we previously described in Murnane et al (2). The weight of the dried solids (TSS) was calculated as:

$$TSS\left[\frac{mg}{mL}\right] = \frac{m_f - m_i}{v_l} \quad \text{(Eqn. S1)}$$

Where m_f is the weight of the dried cells, m_i is the weight of the initial mass of suspended cells, and v_l is the volume of suspended cells taken from growth reactors.

SI4. *Gas chromatography details*

Gas chromatography was used to quantify concentrations of *c*DCE and oxygen in the batch bottles. Specifically, a 6890 Series gas chromatograph (Hewlett Packard, Corvallis, Oregon, United States) equipped with a micro-electron capture device (ECD) was used to quantify the *c*DCE. We separated *c*DCE from other compounds with an Agilent DB-624 UI capillary column (30 m x 0.53 mm) (Agilent Technologies, Santa Clara, California, United States) with ultra-high purity-pure helium (Airgas, Radnor,

Pennsylvania, United States) as the carrier gas (15 mL/min) at 50 ˚C to achieve retention time (RT) of 2.4 min. A 5890 Series GC II (Hewlett Packard, Corvallis, Oregon, United States) equipped with a micro-electron capture device (ECD) was used to measure oxygen levels. We separated oxygen from other compounds with a Supelco 60/80 Carboxen-10000 packed stainless—steel column (15 ft x 1/8 in) (Supelco, Inc., Bellefonte, Pennsylvania, United States) with ultra-high purity-pure helium (Airgas, Radnor, Pennsylvania, United States) as the carrier gas (30 mL min^{-1}), at 40 ˚C, to achieve a retention time (RT) of 4 min. The use of external standards supplied the calibration for all GC methods.

SI5. *Cometabolic and Metabolic experimental data*

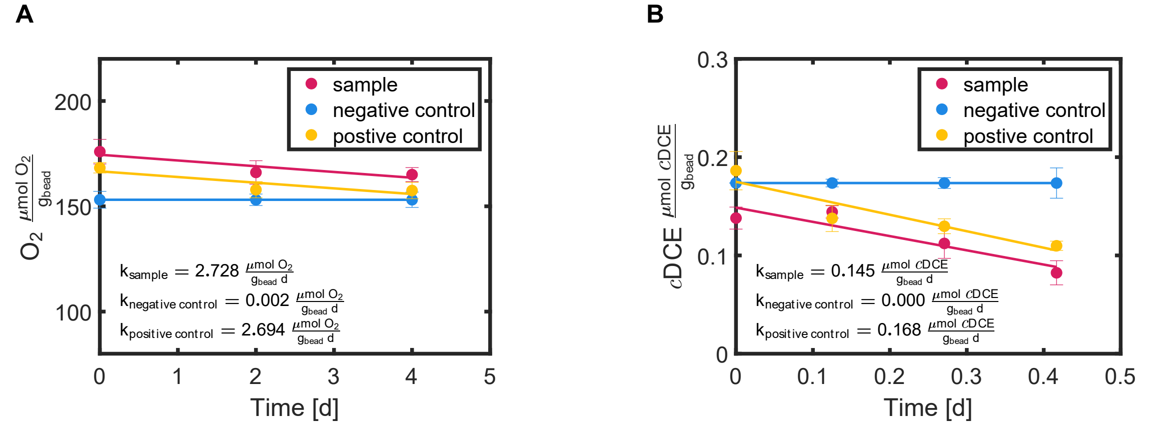

Figure S1 A Typical O_2 uptake data for hydrogel beads obtained from gas chromatography. **B** Typical *c*DCE uptake data for hydrogel beads obtained from gas chromatography. The data was fitted with zero-order rates as data was taken over narrow ranges of time for uptake and transformation and remained linear over the entirety of the time period.

Metabolic activity tests were performed as described in the main text. The beads used in these experiments were formulated with the same formula as the center point of the central composite orthogonal design (Experiment No 15, **Table 2**), following the methods described in the main text (**Methods: Immobilizing ATCC 21198 and TBOS with PVA – AG beads**) and excluding the addition of PVA. Thus, the formula is comprised of 0% (w/v) PVA, 1.5% (w/v) AG, 10% (v/v) TBOS, 0.1% (v/v) Span 80, and 0.5 mg/mL ATCC 21198. Negative control consists of poisoned cells. Gas chromatography (GC) data was used to evaluate the cometabolic and metabolic transformation of *c*DCE and O_2, respectively, with ATCC 21198 immobilized in all bead formulations. See Section 2.4 in the main text for sample preparation and injections. The total mass measured from the GC was taken over time, and zero-order rate laws were applied to the data (**Figure S1A-B**). Note that zero-order rate laws were used in this study as the change in mass over time for both O_2 and *c*DCE was linear for the range of time used to determine the rates.

SI6. ***Hydrogel beads before and after compression tests***

A B

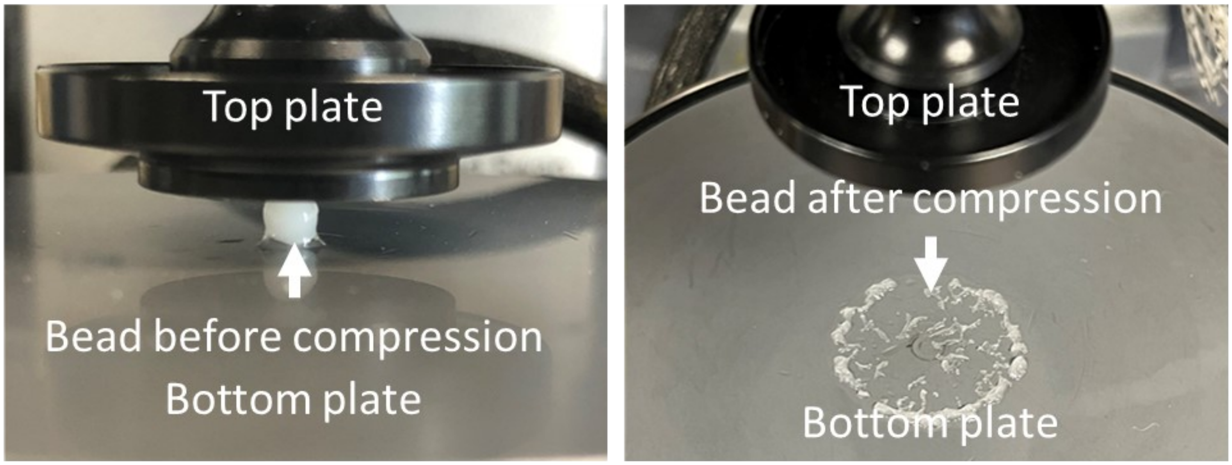

Figure S2 A Hydrogel Bead before compression with top of plate in contact. **B** Hydrogel bead irreversibly deformed after compression test

Hydrogel beads were irreversibly deformed during compression tests. Before compression, beads were slightly compressed such that the axial force in the rheometer exceeded 0.01 N (**Figure S2 A**). The beads

were compressed until the gap reached 50 µm and were completely degraded (**Figure S2 B**).

SI7. ***Compression Experimental data***

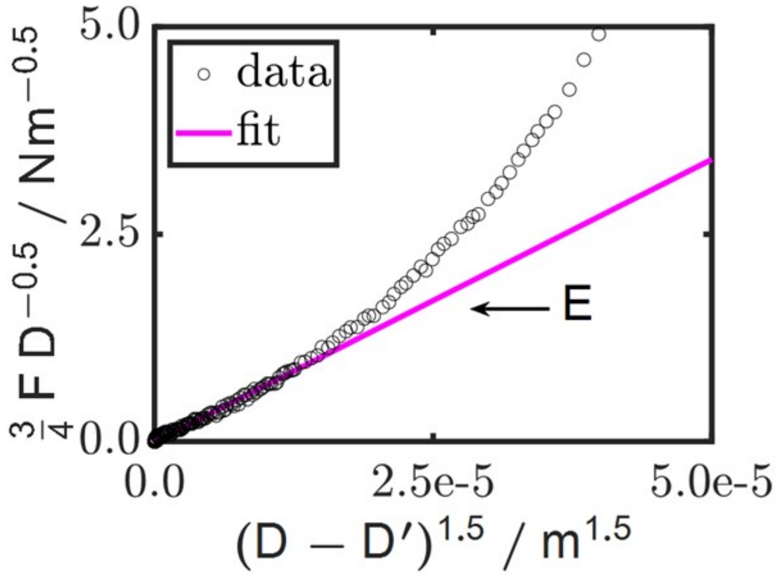

Figure S3 Typical stress-strain data for hydrogel beads obtained from compression tests.

Uniaxial compression measurements were performed on hydrogel beads to obtain the compressive modulus, E (**Figure S3**). The Hertz equation (Eq. 3) can be rearranged and plotted with $3/4\ F\ D^{-0.5}$ against $\Delta D = (D - D')^{1.5}$. The compressive modulus, E, is taken as the slope of the linear portion of the curve between a small range of deformation. After the small deformation ranges, the data transitions to an exponential growth. The linear portion was selected by taking the first point as the start and where the absolute value of the second derivative of the data exceeded 0.01. We approximated the second derivative using the diff() function in MATLAB.

SI8. ***Design of experiments statistical method***

The software MODDE-Pro 12.1 (Sartorius, Fremont, California, United States) was used to generate the CCO experimental matrix and analyze the experimental data obtained. Multiple linear regression was used to obtain predictive models for each response variable. Experimental data was obtained first and used to fit models. Singular value decomposition was used to obtain regression coefficients. Analysis of variance (ANOVA) tests were used to determine the statistical analysis of the

experimental data and regression coefficients, and to obtain interactions between the variables and the responses. A desirability function approach was applied to identify the optimized condition that produced the most desirable responses on dependent variables. The quadratic equations used in DOE for all four dependent variables were:

$$y = \beta_0 + \beta_1 C_{PVA} + \beta_2 C_{AG} + \beta_3 t_{xlink} + \beta_{11} C_{PVA}^2 + \beta_{22} C_{AG}^2 + \beta_{33} t_{xlink}^2 + \beta_{12} C_{PVA} C_{AG} + \beta_{13} C_{PVA} t_{xlink} + \beta_{23} C_{AG} t_{xlink} + \epsilon$$

(Eqn. 10)

where y is the dependent variable, either E_1, E_{30}, $k_{O_2,1}$, or $k_{O_2,1}$; β coefficients represent the overall population value of the response (β_0), the population values for independent variables, and the population values for interactions between the independent variables; and ϵ is the random error of the response. Factor effect plots were used to compare the individual contributions of each factor on each of the responses measured. Each input factor was varied over its specific range while all the other input factors were held constant at their averages. In contrast, response surface maps were used to evaluate the individual and combinatorial effects of multiple input variables on each of the responses measured. Both factor effect plots and response surface maps were generated from the predictive equations for each response as a function of the three input factors.

SI9. ***cis-1,2-dichloroethylene (cDCE) rate data design of experiments models***

We fit *c*DCE rate data on 1 and 30 days ($k_{cDCE,1}$ and $k_{cDCE,30}$) described in the main text under Section 3.1 with second order multivariate regression models and eliminated terms that were not statistically significant. Experimental and predicted data are shown for all bead formulae tested with the central composite design described in the main text (**Table S2**). This data was not used to optimize the bead parameters and therefore was excluded from the main text. The models were evaluated based on the criteria provided in the main text under Section 3.3. That is, the statistical significance of each model was then evaluated with ANOVA tests, with a *p*-value of < 0.05 indicating a significant model and a *p*-value > 0.10 for lack of fit indicating a model with a negligible pure error. We provide the ANOVA assessment for

the $k_{cDCE,1}$ and $k_{cDCE,30}$ regression models to demonstrate that the models were statistically significant and had negligible pure error (**Table S3**). The unscaled models obtained for $k_{cDCE,1}$ and $k_{cDCE,30}$ were:

$$k_{cDCE,1} = -0.15 + 0.26C_{PVA} - 1.9C_{AG} - 0.05t_{xlink} - 0.19C_{PVA}^2 - 0.93C_{AG}^2 + 1 \times 10^{-4}t_{xlink}^2 + 4.1 \times 10^{-3} \text{[illegible]} C_{AG}t_{xlink}$$

(Eqn. S1)

$$k_{cDCE,30} = 0.074 - 0.16C_{AG} - 2.7 \times 10^{-3}t_{xlink}$$ (Eqn. S2)

The factor effects and predictions for $k_{cDCE,1}$ and $k_{cDCE,30}$ regression models are provided to identify the effects of the inputs (C_{PVA}, C_{AG}, t_{xlink}) on these outputs (**Figure S4**). Both C_{PVA} and C_{AG} had a negative quadratic influence on $k_{cDCE,1}$, whereas t_{xlink} had a positively quadratic influence (**Figure S4A**). The maximum point of $k_{cDCE,1}$ was predicted to occur below the midpoint for C_{PVA} ($C_{PVA} < 2$% w/v) and near the midpoint for C_{AG} ($C_{AG} \sim 1.5$% w/v), when t_{xlink} was held constant at $t_{xlink} = 75$ min (**Figure S4B**). For the factor effect plot for $k_{cDCE,30}$, C_{PVA} was shown to have no effect, whereas both C_{AG} and t_{xlink} had a negative linear effect on the response (**Figure S4C**). Thus, the maximum of point of $k_{cDCE,30}$ was predicted to occur at the low value of C_{AG} where t_{xlink} was held constant at $t_{xlink} = 75$ min (**Figure S4D**). While these data could be used to maximize the bioremediation capability, the objective of this research was to maintain strong beads and to ensure that transformation of *c*DCE was not inhibited by the immobilization process. Thus, these models were excluded from the main text.

Table S2 Bead formulae for each experiment, generated by a central composite orthogonal design, plus an additional bead formulation (optimal bead). Experimental and predicted data for $k_{cDCE,1}$ and $k_{cDCE,30}$ are shown for each experimental condition.

				$k_{cDCE,1}$ $[\frac{\mu mol\ cDCE}{g_{bead}\ d}]$		$k_{cDCE,30}$ $[\frac{\mu mol\ cDCE}{g_{bead}\ d}]$	
**No.**	C_{PVA} **[% (w/v)]**	C_{AG} **[% (w/v)]**	t_{xlink} **[min]**	**Exp.**	**Pred.**	**Exp.**	**Pred.**
1	1.0	1.0	30.0	0.15 ± 0.06	0.33	0.65 ± 0.16	0.65
2	3.0	1.0	30.0	0.16 ± 0.03	0.16	0.67 ± 0.17	0.65
3	1.0	2.0	30.0	0.24 ± 0.09	0.23	0.64 ± 0.17	0.49
4	3.0	2.0	30.0	0.05 ± 0.01	0.06	0.36 ± 0.20	0.49
5	1.0	1.0	120.0	0.02 ± 0.01	0.03	0.46 ± 0.04	0.41
6	3.0	1.0	120.0	0.04 ± 0.01	0.03	0.43 ± 0.15	0.41
7	1.0	2.0	120.0	0.12 ± 0.01	0.11	0.28 ± 0.05	0.25
8	3.0	2.0	120.0	0.08 ± 0.02	0.11	0.27 ± 0.04	0.25
9	0.6	1.5	75.0	0.13 ± 0.02	0.13	0.34 ± 0.07	0.45
10	3.4	1.5	75.0	0.04 ± 0.01	0.02	0.21 ± 0.06	0.45
11	2.0	0.8	75.0	0.05 ± 0.01	0.06	0.34 ± 0.04	0.56
12	2.0	2.2	75.0	0.06 ± 0.01	0.04	0.36 ± 0.06	0.34
13	2.0	1.5	14.1	0.15 ± 0.04	0.37	0.15 ± 0.04	0.61
14	2.0	1.5	135.9	0.21 ± 0.08	0.20	0.23 ± 0.02	0.29
15	2.0	1.5	75.0	0.14 ± 0.09	0.15	0.36 ± 0.02	0.45
opt	3.2	2.0	110	0.10 ± 0.01	0.05	0.17 ± 0.02	0.27

175 **Table S3** ANOVA assessment for $k_{cDCE,1}$ and $k_{cDCE,30}$ regression models.

Response	$k_{cDCE,1}$	$k_{cDCE,30}$
**p-values**		
Model	<0.0001	<0.0001
C_{PVA}	0.0031	-
C_{AG}	0.5862	0.0097
t_{xlink}	0.0011	0.0007
C_{PVA}^2	0.0130	-
C_{AG}^2	0.0031	-
t_{xlink}^2	0.0012	-
$C_{PVA}C_{AG}$	-	-
$C_{PVA}t_{xlink}$	0.0095	-
$C_{AG}t_{xlink}$	0.0085	-
Lack of fit	0.8300	0.1760
**Validation Metrics**		
Total Sample Size (N)	15	13
Degree of Freedom (DF)	6	10
R^2	0.93	0.77
R^2-adjusted	0.85	0.72
Q^2	0.65	0.58

176
177

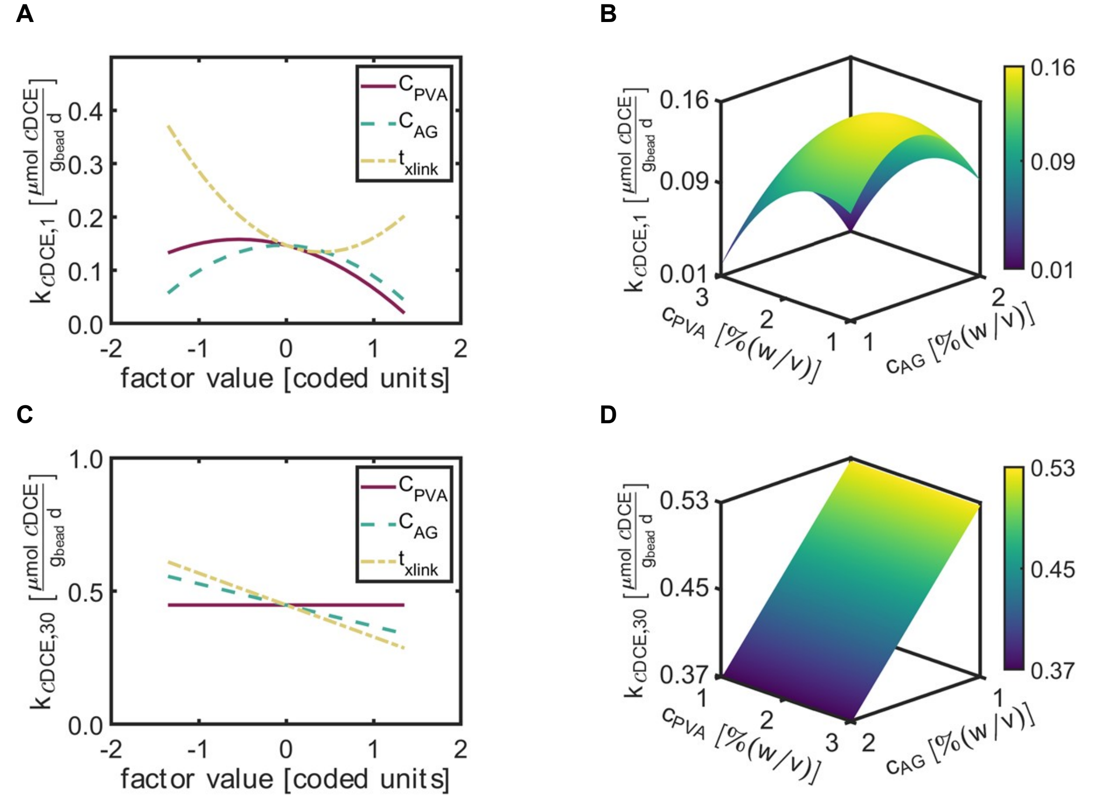

Figure S4 The factor effect plots (left column) include all input variables, C_{PVA}, C_{AG}, and t_{xlink} denoted with red (solid), green (dashed), and yellow (dashed-dotted) lines, respectively. 3-D response surface plots (right column) consist of the output response on the z-axis predicted for a range (factor value $\in [-1, 1]$ / [coded units]) of C_{PVA} and C_{AG} on the x and y-axes, respectively and constant $t_{xlink} = 75$ [min]. The color bar represents the magnitude of the response from low (purple) to high (yellow). **A** Factor effect plot of $k_{cDCE,1}$. **B** 3-D response surface map of $k_{cDCE,1}$. **C** Factor effect plot of $k_{cDCE,30}$. **D** 3-D response surface map of $k_{cDCE,30}$.

SI10. ***Metabolic Activity Control: Rate of oxygen utilization at day 1, alginate only bead controls***

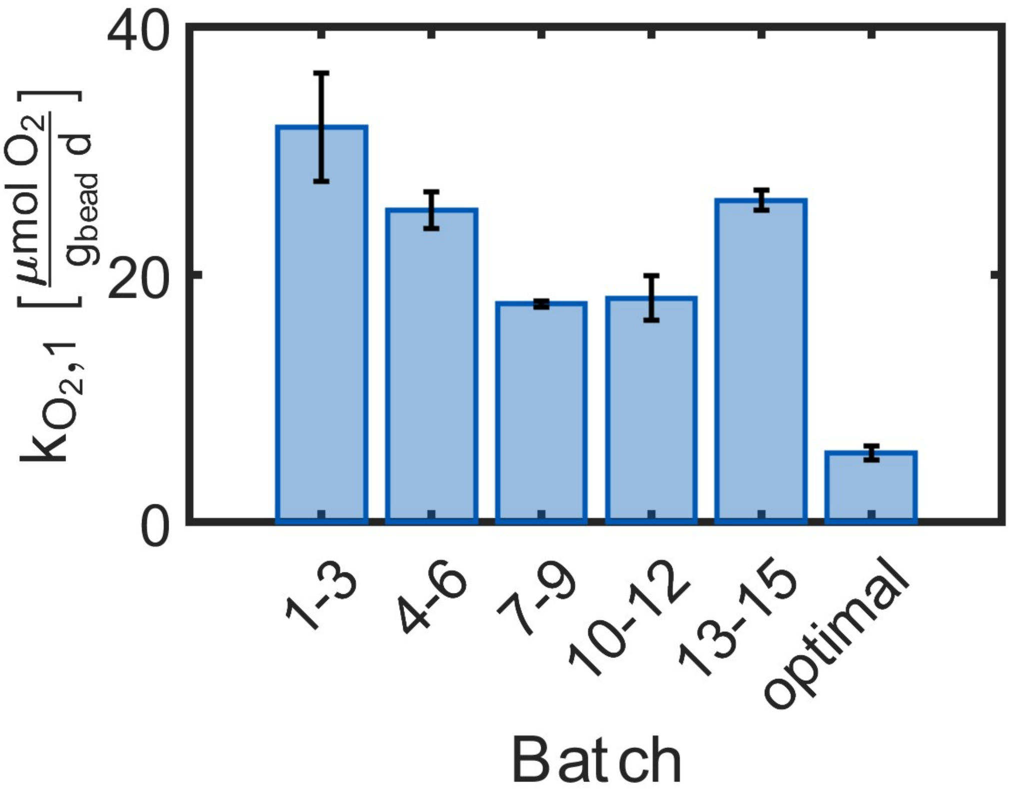

Figure S5. A Rates of oxygen utilization measured for controls for batches made throughout this study. All data are expressed as average ± SD, n = 3. Rates are similar between the first 5 batches (1-3,4-6,7-9,10-12,13-15), but the optimal batch was observed at a much higher rate.

Metabolic activity tests were performed as described in the main text. The beads used in these experiments were formulated with the same formula as the center point of the central composite orthogonal design (Experiment No 15, **Table 2**), following the methods described in the main text (**Methods: Immobilizing ATCC 21198 and TBOS with PVA – AG beads**) and excluding the addition of PVA. Thus, the formula is comprised of 0% (w/v) PVA, 1.5% (w/v) AG, 10% (v/v) TBOS, 0.1% (v/v) Span 80, and 0.5 mg/mL ATCC 21198. 2g of beads were added to 150 mL batch reactors and placed on a shaker table and oxygen was measured on a GC, as described in the main text. The control was made with each batch of beads, indicated by the batch id (1-3, 4-6, 7-9, 10-12, 13-15, optimal). Six controls

were completed throughout the testing with three replicates, each. Between each batch, the oxygen rates for the control bottles were variable (**Figure S5**). Batches 4-6 and 13-15, and batches 7-9 and 10-12 were observed to have similar rates. The optimal batch was measured to have the lowest rate of oxygen utilization rate between all batches, which was likely due to the change in a 90% purity TBOS to a 98% purity TBOS described in the main text (**Section 2.2**). This data suggests that there could be differences between live/dead cells immobilized in the beads.

SI11. *Compression tests under abiotic conditions*

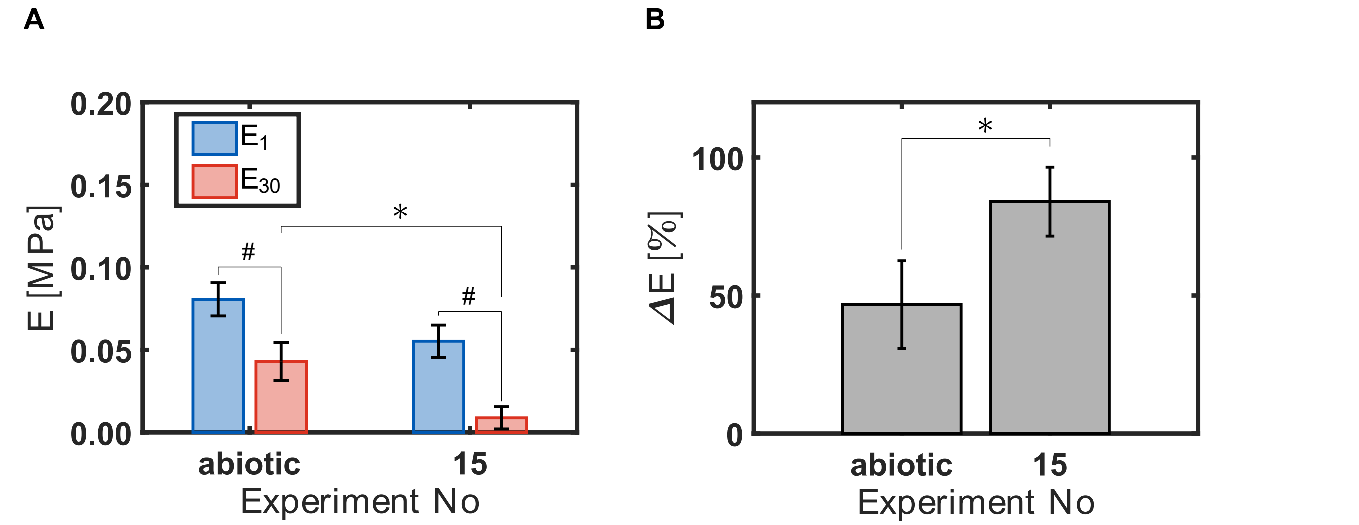

Figure S6 A Compressive modulus E measured for beads under abiotic conditions and center point formulation (Experiment No 15, **Table 2**) at day 1 and day 30. For beads under abiotic conditions, data are expressed as average ± SD, n = 3. For experiment 15, data is expressed as average ± SD, n = 9. * p <0.05 between bead formulations, # p <0.05 between day 1 and day 30 obtained using a two-way ANOVA test with Tukey's honestly significant difference post-test. **B** Elastic loss ΔE for beads under abiotic conditions and center point formulation (Experiment No 15, **Table 2**) at day 1 and day 30. For beads under abiotic conditions, data are expressed as average ± SD, n = 3. experiment 15, data is expressed as average ± SD, n = 9. * *p* <0.05 between bead formulas obtained using a one-way ANOVA test with Tukey's honestly significant difference post-test.

Beads with autoclaved cells were used to evaluate the abiotic degradation of beads. Compression tests were performed as described in the main text. Beads under abiotic conditions were formulated with the same formula as the center point of the central composite orthogonal design (Experiment No 15, **Table**

2), following the methods described in the main text (**Methods: Immobilizing ATCC 21198 and TBOS with PVA – AG beads**) with autoclaved cells. 2g of beads were added to 150 mL batch reactors and placed on a shaker table and removed on day 1 and day 30 for tests with compression, as described in the main text.

For the same bead type, we compared the elastic loss between the beads that were poisoned and beads that were not after 30 days. The abiotic bead was observed to maintain a higher compressive modulus at 30 days when compared to beads with live cells (**Figure S6A**). Moreover, the elastic loss observed for the abiotic bead was near half the elastic loss observed for the beads incubated with live cells (**Figure S6B**). This data suggests that live cells can alter the hydrogel structure and properties, likely due to the proliferation of cells inside the beads.

1. Rolston HM, Hyman MR, Semprini L. Aerobic cometabolism of 1,4-dioxane by isobutane-utilizing microorganisms including Rhodococcus rhodochrous strain 21198 in aquifer microcosms: Experimental and modeling study. Science of The Total Environment. 2019 Dec;694:133688.

2. Murnane RA, Chen W, Hyman M, Semprini L. Long-term cometabolic transformation of 1,1,1-trichloroethane and 1,4-dioxane by Rhodococcus rhodochrous ATCC 21198 grown on alcohols slowly produced by orthosilicates. Journal of Contaminant Hydrology. 2021 Jun;240:103796.
